# Synthesis of Polyanionic C5-Modified 2′-Deoxyuridine and 2′-Deoxycytidine-5′-Triphosphates and Their Properties as Substrates for DNA Polymerases

**DOI:** 10.3390/molecules26082250

**Published:** 2021-04-13

**Authors:** Claire Dutson, Esther Allen, Mark J. Thompson, Joseph H. Hedley, Heather E. Murton, David M. Williams

**Affiliations:** 1 Centre for Chemical Biology, Department of Chemistry, Sheffield Institute for Nucleic Acids, University of Sheffield, Sheffield S3 7HF, UK; claire_dutson@hotmail.com (C.D.); esther.allen@sheffield.ac.uk (E.A.); drmark64@hotmail.co.uk (M.J.T.); 2 QuantuMDx Group, Lugano Building, 57 Melbourne Street, Newcastle upon Tyne NE1 2JQ, UK; Joe_Hedley@hotmail.co.uk (J.H.H.); heathermurton@gmail.com (H.E.M.)

**Keywords:** C5-modified dNTPs, polyanionic reporter group, DNA polymerases

## Abstract

Modified 2′-deoxyribonucleotide triphosphates (dNTPs) have widespread applications in both existing and emerging biomolecular technologies. For such applications it is an essential requirement that the modified dNTPs be substrates for DNA polymerases. To date very few examples of C5-modified dNTPs bearing negatively charged functionality have been described, despite the fact that such nucleotides might potentially be valuable in diagnostic applications using Si-nanowire-based detection systems. Herein we have synthesised C5-modified dUTP and dCTP nucleotides each of which are labelled with an dianionic reporter group. The reporter group is tethered to the nucleobase via a polyethylene glycol (PEG)-based linkers of varying length. The substrate properties of these modified dNTPs with a variety of DNA polymerases have been investigated to study the effects of varying the length and mode of attachment of the PEG linker to the nucleobase. In general, nucleotides containing the PEG linker tethered to the nucleobase via an amide rather than an ether linkage proved to be the best substrates, whilst nucleotides containing PEG linkers from PEG_6_ to PEG_24_ could all be incorporated by one or more DNA polymerase. The polymerases most able to incorporate these modified nucleotides included Klentaq, Vent(*exo*-) and therminator, with incorporation by Klenow(*exo-*) generally being very poor.

## 1. Introduction

Base modified 2′-deoxyribonucleoside-5′-triphosphates (dNTPs) in which a specific reporter group, ligand or catalytic moiety is tethered via a linker to the nucleobase allow the preparation of functionalised nucleic acids that have a wide variety of applications in biotechnology and chemical biology [1]. Typically dNTPs modified at the C5-position of pyrimidines or C7-position of 7-deazapurine bases are good substrates for most DNA polymerases since the modification does not interfere with Watson Crick base pairing. In addition these modifications are in the major groove whilst most protein-DNA interactions during nucleotide incorporation arise in the minor groove [2]. Such modified dNTPs can be used in PCR [1], DNA sequencing [3,4] and labelled for use in structural studies [5]. Other applications of modified dNTPs include the directed evolution of novel ligands and catalysts using SELEX [6,7].

In order to incorporate poorer dNTP substrates, for example with bulkier modifications, a number of groups have evolved existing DNA polymerases to create new polymerases with altered substrate specificities [4,8,9,10]. For example, the Marx group have described [11] the successful incorporation of a dendrimer modified dUTP into an oligodeoxynucleotide (ODN) using KlenTaq DNA polymerase and a C5-ODN-modified dUTP using Therminator DNA polymerase [12]. They observed that the rigid alkyne-based C5-anchor group was sufficient to hold the more flexible dendrimer and ODN side chains outside of the active sites of the polymerases. Typically, for dNTPs involving C5-modified pyrimidines, having a rigid linker (typically containing an alkyne or alkene) tethering the modification to the nucleobase are better substrates for DNA polymerases [13,14].

There are relatively few examples of pyrimidine-modified dNTPs bearing negatively charged reporter groups in the literature and this is further restricted when examining dCTP analogues. These types of modified dNTPs would increase the potential for accessing novel functionalized nucleic acids.

Our interest in these types of nucleotides derives from their potential use in the diagnostic analysis of nucleic acids using Si-nanowire-based biosensors [15]. In this latter context nucleic acid hybridization to a Si-nanowire immobilized PNA changes the charge density and resistance of the nanowire, thereby producing a biosensing signal [16,17,18,19]. Monitoring the incorporation of suitably charge-modified dNTPs using such devices might therefore allow sequence determination. Here we describe the design and synthesis of C5-pyrimidine modified dNTPs bearing polyanionic side chains and evaluate their substrate properties for a variety of DNA polymerases.

## 2. Results and Discussion

**Figure 1:**
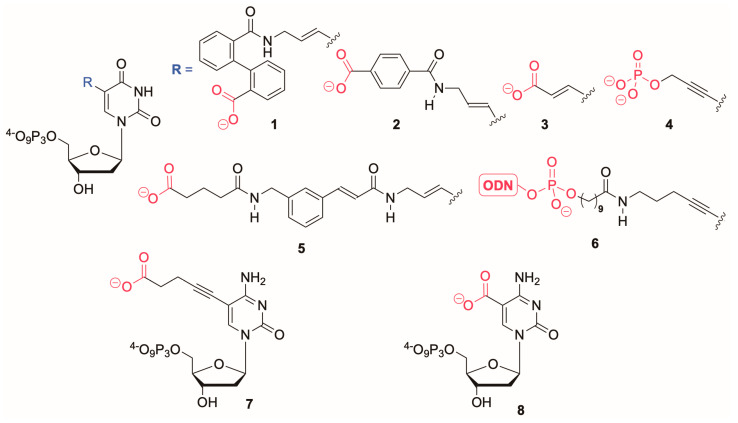
Examples of base modified dNTPs with negatively charged side chains (ODN = oligodeoxyribonucleotide).

There are relatively few examples of C5-modified dUTP analogues with negatively charged side chains in the literature (Figure 1). Of these, the dUTP analogues **1** and **2** could not be incorporated into DNA by polymerases [20]. Compound **3** is a substrate for KOD XL DNA polymerase [21] and in common with **4**, can be incorporated by PrimeSTAR HS and Pwo polymerases [22]. The dNTP **5** is a substrate for Taq DNA polymerase [21], whilst nucleotides of general structure **6**, which have ODNs of differing length tethered to the base are incorporated into DNA by the engineered polymerase KlenTaq [12]. In the dCTP series, the 5-valeric acid-dCTP **7** has been successfully incorporated [23] during primer extension reactions by Vent (*exo-*), Pwo and 9°N_m_ DNA polymerases in addition to the Klenow fragment of DNA polymerase I. Successful amplification during PCR was also demonstrated. The modified dCTP **8**, can be incorporated into DNA using KOD XL DNA polymerase [24]. These studies indicate that the substrate properties of these modified nucleotides depend upon the type and/or length of the linker between the nucleobase and charged moiety: Specifically, with a suitably long linker, even bulky substituents can be tolerated.

**Figure 2:**
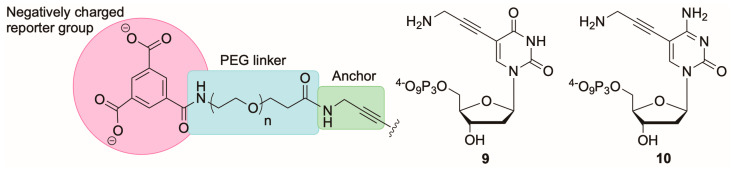
Linker design for C5-modified dUTP and dCTP analogues (left) and structures of precursor C5-propargylamino-modified nucleotides (**9**) and (**10**).

Influenced by the findings of previous studies, we designed a series of modified dNTPs with three features; a rigid C5 anchor attached to the C5 atom of the nucleobase, a flexible PEG based linker of varying length and a charged reporter moiety (Figure 2).

We chose to use trimesic acid as the anionic reporter group that would be tethered to the C5 positions of the triphosphates **9** and **10** using polyethyleneglycol (PEG) linkers of differing lengths. PEG linkers were chosen in order to maintain good water solubility of the dNTPs. To obtain the desired nucleotides we required PEG linkers functionalized with amino and carboxylate termini (CA-PEG) that would facilitate coupling to the reporter group and nucleotides via amide coupling reactions. Thus, commercially available PEG linkers CA-PEG_8_ and CA-PEG_24_ were initially chosen in order to fully explore the effect of linker length on the substrate properties of the dNTPs. These PEG linkers give approximate lengths [25] of 2.9 nm (PEG_8_) to 8.6 nm (PEG_24_) i.e., distances of around 9 or 25 base pairs in DNA.

**Scheme 1:**
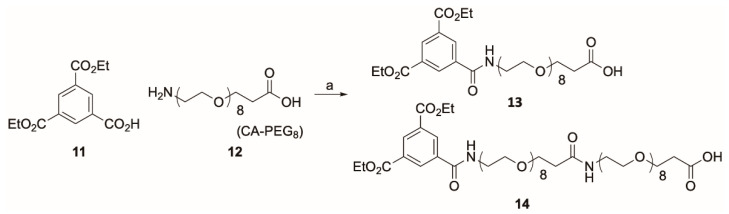
HATU coupling reaction of diethyl 1,3,5-benzenetricarboxylate (**11**) with CA-PEG_8_ forming the desired product **13** (31% yield) and the extended product **14** (19% yield). *Reagents and conditions*: Dry DMF, HATU, DIPEA, **11**, CA-PEG_8_ (**12**), 0 °C to r.t. over 30 min.

To prepare the desired reporter-linker units we attempted a HATU-mediated amide coupling between the diester of trimesic acid (**11**) and commercially available CA-PEG_8_ (**12**)**.** However, this formed not only the desired linker-reporter unit **13**, but also the extended product **14** (Scheme 1) that were isolated as colourless oils in 31% and 19% yield, respectively, following reversed phase HPLC.

**Scheme 2:**
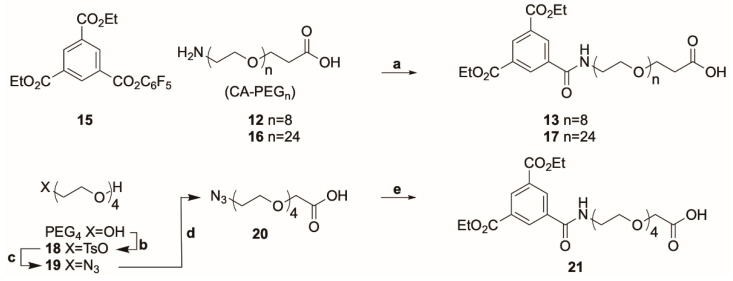
Synthesis of linker-reporter units using from diethyl 1,3,5-benzenetricarboxylate penTable 13. (61%), **17** (73%); (**b**) TsCl, Et_3_N, r.t., 92%; (**c**) NaN_3_ in EtOH, 70 °C, 79%; (**d**) NaH, THF, BrCH_2_CO_2_H, r.t., 88%; (**e**) H_2_, 10% Pd-C in MeOH, 50 °C, 30 bar, then **15**, DMF, DIPEA, r.t., 25% (two steps).

Consequently, in order to improve the yield of **13** we chose an alternative route in which we converted the acid **11** to its corresponding pentafluorophenyl ester **15**. Compound **15** in turn could be reacted cleanly with CA-PEG_8_ (**12**) to afford **13** in 61% yield following RP-HPLC (Scheme 2). CA-PEG_24_ (**16**) was conjugated in the same manner to afford **17** and the analogous PEG_4_ compound **21** was synthesized from PEG-4 (Scheme 2).

**Figure 3:**
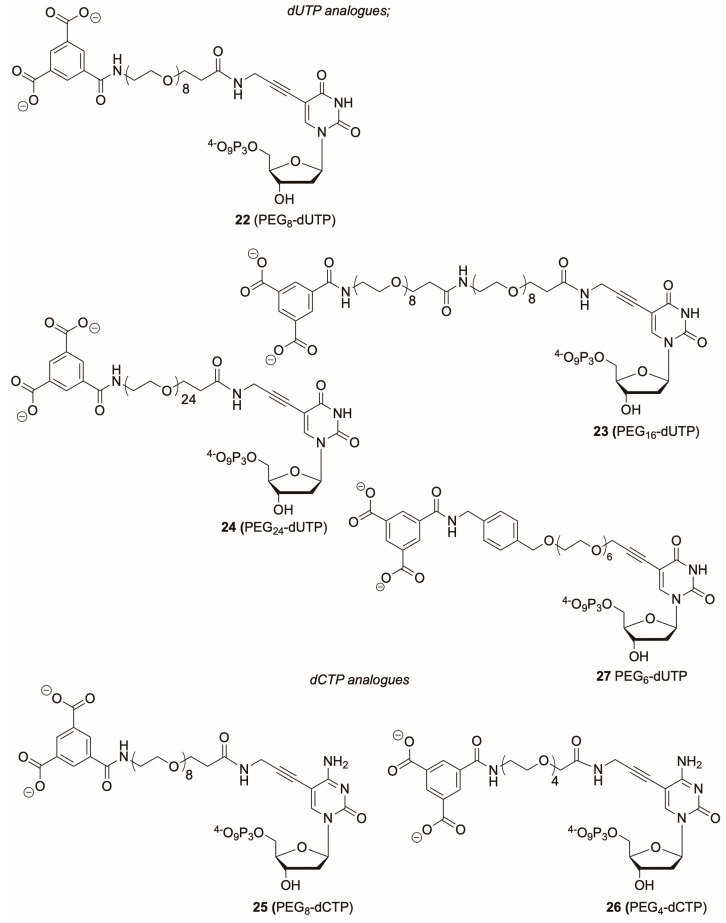
Structures of modified dNTPs.

**Scheme 3:**
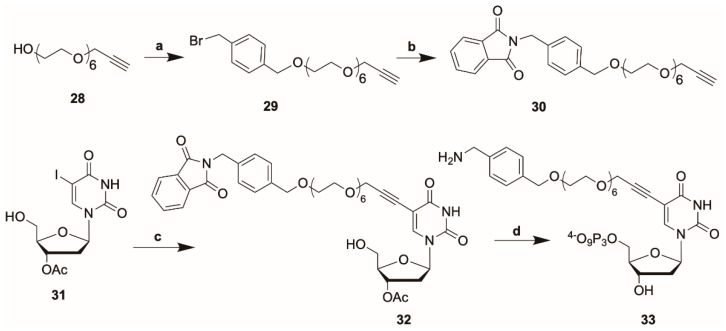
Synthesis of compound **33** (precursor to dUTP **26**). *Reagents and Conditions*: (**a**) NaH, DMF, 1,4-dibenzylbromide, 42%; (**b**) potassium phthalimide, DMF, 100 °C, 86%; (**c**) 31, CuI, Pd(PPh_3_)_4_, Et_3_N, DMF, r.t., 38%; (**d**) salicyl chlorophosphite, P_2_O_7_^4−^, then I_2_ aq pyridine, then MeNH_2_, 35%.

The reporter-linker units **13**, **14**, **17** were then conjugated to C5-propargylamino-dUTP (**9**) [13] using TSTU as the coupling agent. The products were then treated with aqueous sodium hydroxide solution followed by RP-HPLC to afford the modified dNTPs **22**–**24** (Figure 3), typically in yields around 60%. Analogous reactions of **13** and **21** with C5-propargylamino-dCTP (**10**) [21] gave modified dCTPs **25** and **26**, respectively. Alongside these dNTP analogues we also prepared the dUTP analogue **27**. This contains an *O*-propargyl-PEG linker. C5-modified dUTPs with an *O*-propargyl-PEG_3_ linker are known to be substrates for KlenTaq DNA polymerase. [26] The linker unit for dUTP **27** was prepared from the *O*-propargyl-PEG_6_ compound **28** which was reacted with 1,4-(dibromomethyl)benzene to give **29** which was then reacted with potassium phthalimide to give **30**. A Sonogashira coupling between compound **30** and 3′-*O-*acetyl-5-iodo-2′-deoxyuridine (**31**) [27] provided the modified nucleoside **32** (Scheme 3). Phosphorylation of nucleoside using the Ludwig-Eckstein method [28] followed by treatment with aqueous methylamine solution provided dUTP **27** in 35% yield.

**Figure 4:**
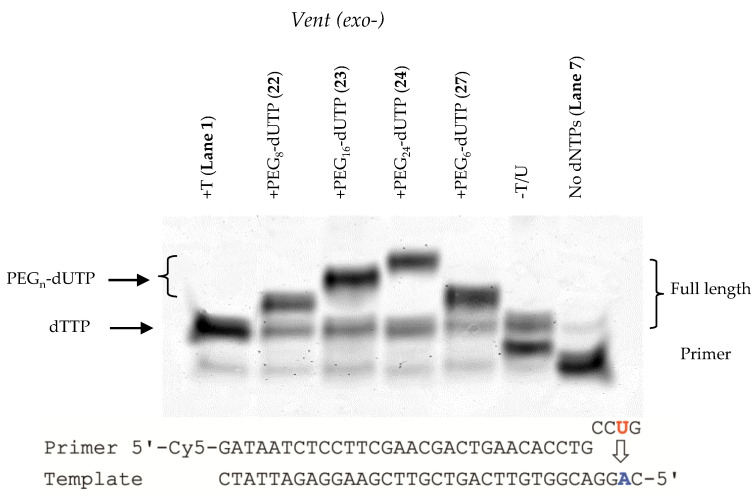
PAGE gel for the incorporation of PEG modified dUTPs with the use of Vent (*exo-*) DNA polymerase. All lanes except 7 contain dATP, dCTP, dGTP and the nucleotide indicated above the gel (from left): **Lane 1** = dTTP, dATP, dCTP, dGTP; **Lane 2** = PEG_8_-dUTP (**22**), dATP, dCTP, dGTP; **Lane 3** = PEG_16_-dUTP (**23**), dATP, dCTP, dGTP; **Lane 4** = +PEG_24_-dUTP (**24**), dATP, dCTP, dGTP; **Lane 5** = PEG_6_-dUTP (**27**), dATP, dCTP, dGTP; **Lane 6** = dATP, dCTP, dGTP; **Lane 6** = Control, no dNTPs present. Conditions for all primer reactions; 20 nM primer, 15 nM template, 625 nM dNTPs, Vent (*exo-*) DNA polymerase, 40 °C, 1 h.

To compare the effects of the linker on the substrate properties of the modified nucleotides, we first investigated the incorporation of the C5-modified dUTPs in primer extension reactions using Vent (*exo*-) DNA polymerase (Figure 4). Initially we chose a simple template containing the nucleotide sequence AC requiring insertion of dTTP/dUTP and dGTP. Each reaction contained dATP, dCTP and dGTP together with dTTP or one of the four dUTP nucleotides (**22**, **23**, **24** and **27**). Full length products were observed in each case. When dTTP or dUTP was absent (i.e., using only dATP, dCTP and dGTP) significant amounts of n + 1 product were observed (Figure 4, lane 6). In contrast, incorporation of the modified nucleotides results in a significantly decreased gel mobility of the extended products that was dependent on the molecular weight of the linker (Figure 4, lanes 2–5). Furthermore, the nature of the PEG linker (propargylamido (**22**–**24**) or *O*-propargyl (**27**)) did not appear to affect properties of the modified dUTPs as substrates for Vent (*exo*-).

**Figure 5:**
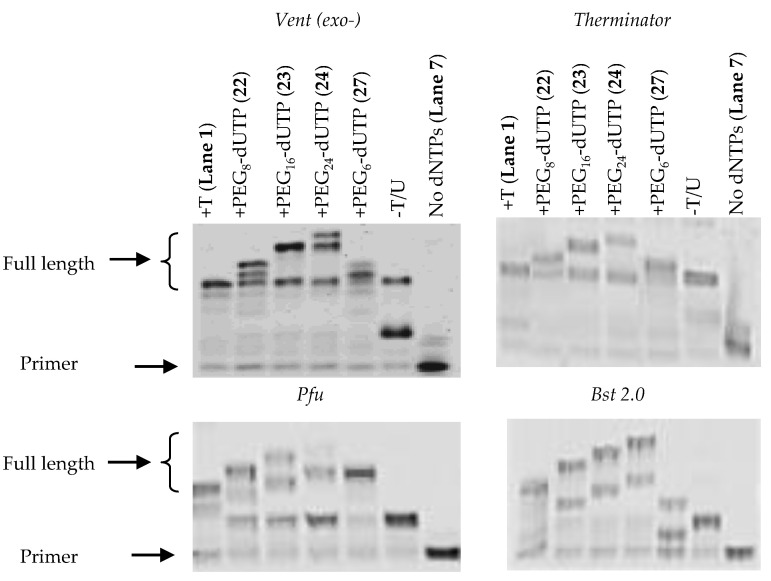
PAGE analysis of the incorporation of PEG modified dUTPs with the use of Vent (*exo-*), Therminator, Pfu and Bst 2.0 DNA polymerases. All lanes except “no dNTPs” contain dATP, dCTP, dGTP and the nucleotide indicated above the gel (from left): **Lane 1** = dTTP, dATP, dCTP, dGTP; **Lane 2** = PEG_8_-dUTP (**22**), dATP, dCTP, dGTP; **Lane 3** = PEG_16_-dUTP (**23**), dATP, dCTP, dGTP; **Lane 4** = PEG_24_-dUTP (**24**), dATP, dCTP, dGTP; **Lane 5** = PEG_6_-dUTP, dATP, dCTP, dGTP; **Lane 6** = dATP, dCTP, dGTP; **Lane 7** = Control, no dNTPs present. Conditions for all primer reactions; 20 nM primer, 15 nM template, 625 nM dNTPs, 40 °C, 1 h.

In order to investigate the ability of DNA polymerases to extend from the modified nucleotides following incorporation, we chose a different primer-template requiring the insertion of the modified nucleotide followed by incorporation of a further 10 natural dNTPs (Figure 5). In addition, we included the polymerases Bst 2.0, Therminator and Pfu. In reactions using Vent (*exo-*) polymerase nucleotide incorporation and extension can be observed, however in each case there appears to be some misincorporation of the the natural nucleotides indicated by the presence of faster-mobility full-length product obersved by PAGE. A similar outcome is observed using Therminator polymerase. However, in each case the reduced mobility of the full-length products indicates the incorporation of the modified nucleotides **22**–**24** and their subsequent extension albeit with some amounts of misincorporation of the natural nucleotides. When the polymerases Bst 2.0 and Pfu were examined, two product bands were observed by PAGE, indicative of the primer extension reaction partially stalling after the incorporation of the modified dUTPs. The results also show that the after incorporation of PEG_6_-dUTP (**27**), which contains a propargyl ether in the linker, further extension by Bst 2.0 was not possible.

**Figure 6:**
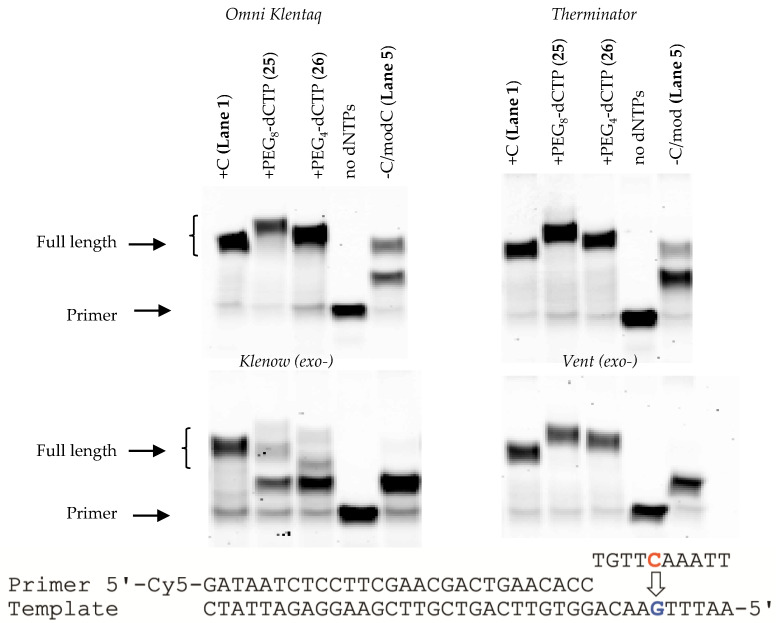
PAGE analysis of the incorporation of PEG modified dCTPs with the use of Omni-Klentaq, Therminator, Klenow (*exo-*) and Vent (*exo-*) DNA polymerase. All lanes except “no dNTPs” contain dATP, dGTP, dTTP and the nucleotide indicated above the gel (from left): **Lane 1** = dCTP, dATP, dGTP, dTTP; **Lane 2** = PEG_8_-dCTP (**25**), dATP, dGTP, dTTP; **Lane 3** = PEG_4_-dCTP (**26**), dATP, dGTP, dTTP; **Lane 4** = dATP, dGTP, dTTP; **Lane 5** = Control, no dNTPs present. Conditions for all primer reactions; 20 nM primer, 15 nM template, 625 nM dNTPs, 40 °C, 1 h.

We then examined incorporation of the dCTP analogues PEG_4_-dCTP (**26**) and PEG_8_-dCTP (**25**). Since Vent (*exo-*) and Therminator had shown incorporation of the modified dUTPs and subsequent extension of the primer, we chose both of these polymerases to examine their incorporation together with a further two DNA polymerases, Omni Klentaq and Klenow (*exo-*). All polymerases with the exception of Klenow (*exo-*) successfully incorporated the modified dCTPs and subsequently extended the primer (Figure 6). However misincorporation can still be observed with Therminator and Omni Klentaq. Although Klenow (*exo-*) did not successfully incorporate either modified dCTP within the 1 h incubation period with an extended incubation period of 7 h some incorporation and extension was seen (data not shown).

In summary, we have described the chemical synthesis of C5-modified dUTP and dCTP analogues functionalized with anionic reporter group tethered via different length PEG-based linkers. We have shown that these analogues can be incorporated by a variety of different polymerases producing full-length products with a significantly decreased gel mobility. We anticipate that these functionalized nucleotides will have a variety of applications in biotechnology and will report further on these in due course.

## 3. Materials and Methods

### 3.1. General Methods

Dry solvents were obtained from a Grubbs dry solvent apparatus. Column chromatography purifications were carried out on silica (60–200 mesh, VWR Chemicals, Hayes, UK). Thin layer chromatography (TLC) was performed on pre-coated silica gel 60 F254 aluminium backed plates (Merck, London, UK). TLCs were visualised under UV (254 nm). NMR spectra were recorded on either a Bruker AV250, AV400 or AV500 spectrometer (as individually stated for all data) (Bruker, Billerica, MA, USA) and chemical shifts are reported in δ values relative to tetramethylsilane as an external standard. *J* values are given in Hz. All ^1^H, ^13^C and ^31^P spectra are available as supplementary materials. Mass spectrometry was performed by the University of Sheffield Mass Spectrometry Service using the method of electrospray ionisation on an LCT Mass Spectrometer (Waters, Millford, MA, USA) unless otherwise stated. Analytical RP-HPLC was performed on Waters 2695 or 2690 instrumentation using a Gemini C18 5 μm 4.6 × 250 mm column (Phenomenex, London, UK) at a flow rate of 1 mL/min, UV detection was accomplished at 260 nm unless specified otherwise. Preparative RP-HPLC was performed using a Phenomenex Gemini C18 5 μm 110Å 21.2 × 250 mm column at a flow rate of 21 mL/min. UV detection was recorded at 260 nm unless specified otherwise. All quoted retention those found by analytical RP-HPLC. 3,5-Diethyl-1,3,5-benzenetricarboxylate (**11**) was purchased from Sigma-Aldrich (London, UK) and CA-PEG_8_ (12) CA-PEG_24_ (**16**) were purchased from ThermoFisher (Waltham, MA, USA). Compounds **9** and **10** were synthesized as previously described [13,21] and bis(tri-*n*-butylammonium) pyrophosphate was also prepared as described [28].

### 3.2. Chemical Synthesis

#### 3.2.1. (3,5-Diethoxycarbonyl-1-benzenecarbonyl)-CA-PEG_8_ (**13**) 

CA-PEG_8_ (**12**, 500 mg, 1.13 mmol) and 1,3-diethyl-5-pentafluorophenyl-1,3,5-benzenetricarboxylate (**15**, 587 mg, 1.35 mmol) were added to a dried flask. Anhydrous MeOH (10 mL) and dry diisopropylethylamine (200 µL, 1.13 mmol) were added and the reaction stirred at r.t. for 20 h. The crude reaction mixture was analysed by analytical RP-HPLC (100% A for 25 min; ramp to 95% B over 1 min; hold 5 min; to 100% A over 1 min; hold 8 min (A = 60% H_2_O in MeCN + 0.1% TFA, B = MeCN) which showed the product at 14 min retention time. Subsequent purification by preparative RP-HPLC using the same gradient gave the product (550 mg, 61%). *R*_f_ (10% EtOAc in hexane) 0.2; ^1^H-NMR (400 MHz, CD_3_OD) δ_H_ 8.76 (s, 1H, phenyl CH), 8.71 (s, 2H, 2 × phenyl CH), 4.45 (q, *J* = 7.1 Hz, 4H, 2 × OCH_2_CH_3_), 3.87–3.48 (m, 34H, OCH_2_CH_2_), 2.56 (t, *J* = 6.2 Hz, 2H, CH_2_C = O), 1.45 (t, *J* = 7.1 Hz, 6H, 2 × OCH_2_CH_3_) ppm; ^13^C-NMR (100.62 MHz, CDCl_3_) δ_C_ 166.0, 165.2, 134.8, 133.1, 132.3, 131.4, 70.5, 70.4, 70.3, 69.7, 66.6, 61.7, 51.7, 50.8, 40.2, 34.8, 14.3 ppm; HR ES-MS *m/z* calcd for C_32_H_51_NO_15_ [M + H]^+^ 690.3337, found 690.3357.

#### 3.2.2. (3,5-Diethoxycarbonyl-1-benzenecarbonyl)-CA-PEG_16_ (**14**) 

Diethyl 1,3,5-benzenetricarboxylate (**11**, 526 mg, 1.97 mmol) was dissolved in anhydrous DMF (15 mL) under N_2_, HATU (951 mg, 2.50 mmol) added and the mixture cooled to 0 °C. DIPEA (0.89 mL, 5.2 mmol) was then added and the mixture stirred for 30 min. CA-PEG_8_ (**12**, 1.00 g, 2.27 mmol) was then added and the mixture allowed to warm to r.t. over 5 h. The mixture was then evaporated to dryness and the residue purified by prep RP-HPLC using 40% MeCN/water containing 0.1% TFA as the mobile phase. Two colourless oils were obtained after collection and evaporation: peak A at retention time 12.2 min and peak B with retention time 12.8 min. Peak A was identified as compound **14** (407 mg, 0.37 mmol, 19%) and peak B as compound **13** (422 mg, 0.61 mmol, 31%). Data for **13** as described above. Data for **14**: ^1^H-NMR (400 MHz, D_2_O) δ_H _ 8.21 (s, 2H, 2 × phenyl CH), 8.19 (s, 1H, phenyl CH), 4.21 (q, *J* = 7.1 Hz, 4H, 2 × OCH_2_CH_3_), 3.65–3.35 (m, 64H, OCH_2_CH_2_), 3.20 (t, *J* = 6.0 Hz, 2H, CH_2_CO2H), 2.45 (t, *J* = 6.2 Hz, 2H, CH_2_NHCO), 2.35 (t, *J* = 6.2 Hz, 2H, CH_2_N), 1.22 (t, *J* = 7.1 Hz, 6H, 2 × OCH_2_CH_3_) ppm; ^13^C-NMR (100.62 MHz, D_2_O) δ_C_ 175.97, 173.96, 167.45, 166.12, 134.41, 132.59, 132.06, 130.74, 69.55, 69.40, 68.77, 66.70, 66.11, 62.73, 39.73, 38.94, 35.91, 34.25, 13.43; HR ES-MS *m/z* calcd for C_51_H_88_N_2_O_24_ [M + H]^+^ 1112.5727, found 1112.5745.

#### 3.2.3. 1,3-Diethyl-5-(pentafluorophenyl)-1,3,5-benzenetricarboxylate (**15**) 

Diethyl 1,3,5-benzenetricarboxylate (**11**, 1.20 g, 4.50 mmol) was dissolved in anhydrous DMF (20 mL) under N_2_ and EDCI (950 mg, 4.95 mmol) added. Pentafluorophenol (910 mg, 4.95 mmol) was dissolved in anhydrous DMF (5 mL) and added slowly to the reaction mixture. After stirring for 40 min., the solvent was evaporated and the crude residue dissolved in acetone (100 mL). To this was added pH 6.2 phosphate buffer (50 mL of a 0.01 M solution). The solution was extracted with EtOAc (3 × 50 mL) and the combined organic layers dried (MgSO_4_) and concentrated to give a clear oil. The crude product was purified by silica column chromatography (eluent 10% EtOAc in hexane) to give a white solid (1.48 g, 76%). *R*_f_ (10% EtOAc in hexane) 0.38; ^1^H-NMR (250 MHz, CDCl_3_) δ_H_ 9.02 (s, 3H, phenyl CH), 4.49 (q, *J* = 7 Hz, 4H, 2 × CH_2_), 1.47 (t, *J* = 7 Hz, 6H, 2 × CH_3_) ppm; ^13^C-NMR (100.62 MHz, CDCl_3_) δ_C_ 164.5, 161.3, 142.5, 141.1, 140.0, 139.3, 138.5, 136.7, 136.1, 135.3, 132.2, 128.0, 62.0, 14.3 ppm; HR ES-MS *m*/*z* calcd for C_19_H_14_O_6_F_5_ [M + H]^+^ 433.0711, found 433.0729.

#### 3.2.4. (3,5-Diethoxycarbonyl-1-benzenecarbonyl)-CA-PEG_24_ (**17**) 

CA-PEG_24_ (**16**, 100 mg, 87 µmol) and pentafluorophenyl ester (**15**) (45 mg, 105 µmol) were added to a dried flask. Anhydrous MeOH (2 mL) and dry diisopropylethylamine (15 µL, 87 µmol) were added and the reaction stirred at r.t. for 18 h. The crude reaction mixture was analysed by analytical RP-HPLC (100% A for 25 min; ramp to 95% B over 1 min; hold 5 min; to 100% A over 1 min; hold 8 min (A = 60% H_2_O in MeCN + 0.1% TFA, B = MeCN), UV detection at 254 nm) which showed the product at 11 min retention time. Subsequent purification by preparative RP-HPLC using the same gradient gave the product (88 mg, 73%). *R*_f_ (10% EtOAc in hexane) 0.1; ^1^H-NMR (400 MHz, CD_3_OD) δ_H_ 8.76 (s, 1H, phenyl CH), 8.72 (s, 2H, 2 × phenyl CH), 4.48 (q, *J* = 7.1 Hz, 4H, 2 × OCH_2_CH_3_), 3.86–3.49 (m, 98H, OCH_2_CH_2_), 2.57 (t, *J* = 6.2 Hz, 2H, CH_2_C=O), 1.46 (t, *J* = 7.1 Hz, 6H, 2 × OCH_2_CH_3_) ppm; ^13^C-NMR (100.62 MHz, CD_3_OD) δ_C_ 165.0, 132.3, 132.0, 131.4, 70.1, 69.0, 66.4, 61.5, 39.8, 34.4, 13.2 ppm; HR ES-MS *m*/*z* calcd for C_64_H_116_NO_31_ [M + H]^+^ 1394.7531, found 1394.7462.

#### 3.2.5. 1-(4-Methylbenzenesulfonyl)-3,6,9-trioxaundecane-11-ol (**18**) 

Triethylamine (54 mL, 0.39 mol) was added to a stirred solution of tetraethylene glycol (50 g, 0.26 mol) in anhydrous DCM (20 mL). The reaction was cooled to 0 °C, freshly recrystallised 4-toluenesulfonyl chloride (4.9 g, 25.7 mmol) added and the mixture stirred at r.t. overnight. The solvent was then evaporated to give an oil that was dissolved in DCM (300 mL) and washed with water (3 × 20 mL). The organic layer was then washed with acetic acid (0.1 M, 30 mL), water (30 mL), dried (MgSO_4_) and evaporated to give a clear oil (8.25 g, 92%). Analytical data corresponded to that described previously [29].

#### 3.2.6. 1-Azido-3,6,9-trioxaundecane-11-ol (**19**) 

Sodium azide (3.85 g, 59.2 mmol) was added portionwise to a stirred solution of compound **19** (8.25 g, 23.7 mmol) in ethanol (100 mL) over a period of 15 min. Once homogeneous, the reaction was heated to 70 °C. After 20 h. the reaction was cooled to r.t. and the mixture then evaporated to dryness to give a orange oil that was dissolved in DCM (100 mL), washed with water (3 × 30 mL), and the organic layer dried (MgSO_4_) and evaporated to give a pale yellow oil (4.1 g, 79%); ^1^H-NMR (250 MHz, CDCl_3_) δ_H_ 3.74–3.55 (m, 14H, 7 × CH_2_), 3.38 (t, *J* = 5.0 Hz, 2H, CH_2_), 2.78 (bs, 1H, OH); ^13^C-NMR (63 MHz, CDCl_3_) δ_C_ 72.48, 70.65, 70.62, 70.55, 70.30, 70.01, 61.65, 50.62; HR ES-MS *m*/*z* calcd for C_8_H_17_N_3_O_4_ [M + H]^+^ 220.1292, found 220.1289.

#### 3.2.7. 14-Azido-3,6,9,12-tetraoxatetradecan-1-oic Acid (**20**) 

Sodium hydride (2.46 g, 61.5 mmol) was added portionwise to a stirred solution of compound **19** in anhydrous THF (60 mL) at 0 °C over a period of 30 min. Once in solution, bromoacetic acid (3.56 g, 25.6 mmol) was added and the reaction stirred overnight at r.t. The reaction was then quenched with the slow addition of water (5 mL) and stirred for 15 min. The solvent was removed under reduced pressure and the residue dissolved in DCM (150 mL), washed with HCl (2 M, 50 mL), sat. brine (50 mL) and dried (MgSO_4_) before being concentrated to give the title compound as a light orange oil (5.52 g, 88%); ^1^H-NMR (250 MHz, CDCl_3_) δ_H_ 4.15 (s, 2H, CH_2_), 3.77–3.70 (m, 2H, CH_2_), 3.69–3.62 (m, 12H, 6 × CH_2_), 3.37 (t, *J* = 5.0 Hz, 2H, CH_2_); ^13^C-NMR (63 MHz, CDCl_3_) δ_C_ 173.18, 71.16, 70.62, 70.56, 70.49, 70.29, 69.96, 68.62, 50.59, 29.65; HR ES-MS *m*/*z* calcd for C_10_H_19_N_3_O_6_ [M + H]^+^ 278.1347, found 278.1350.

#### 3.2.8. (3,5-Diethoxycarbonyl-1-benzenecarbonyl)-CA-PEG_4_ (**21**) 

Compound **20** (0.30 g, 1.08 mmol) in MeOH (10 mL) was placed under a H_2_ atmosphere (30 bar) at 50 °C using a ThalesNano H-Cube (Budapest, Hungary). The solution was then passed over a 10% Pd/C Catcart^®^ at 1 mL/min. The solvent was removed under reduced pressure to give the title compound as pale yellow oil (0.25 g). The oil was then dissolved in in anhydrous DMF (10 mL) and dry diisopropylethylamine (170 µL, 1 mmol) followed by compound **15** (0.52g, 1.2 mmol) and the reaction stirred at r.t. overnight. Evaporation of the solvent and purification by prep-RP-HPLC gave a clear oil (0.11 g, 25%). HPLC gradient: 20–70% B over 20 min where A = H_2_O + 0.1% trifluoroacetic acid and B = MeCN + 0.1% trifluoroacetic acid. Retention time of product = 10.5 min; ^1^H-NMR (250 MHz, CDCl_3_) δ_H_ 8.80 (t, *J* = 1.6 Hz, 1H, Ar-CH), 8.70 (d, *J* = 1.6 Hz, 2H, 2 × Ar-CH), 7.57 (bs, 1H, NH), 4.44 (q, *J* = 7.1 Hz, 4H, 2 × CH_2_), 4.16 (s, 2H, CH_2_), 3.78–3.59 (m, 16H, 8 × CH_2_), 1.44 (t, *J* = 7.1 Hz, 6H, 2 × CH_3_); ^13^C-NMR (101 MHz, CDCl_3_) δ_C_ 173.05, 165.40, 134.60, 133.39, 132.48, 131.40, 70.79, 70.27, 70.06, 69.67, 68.59, 61.94, 40.37, 14.20; HR ES-MS *m*/*z* calcd for C_23_H_33_NO_11_ [M + H]^+^ 500.2116, found 500.2132.

#### 3.2.9. [(3,5-Dicarboxy-1-benzenecarbonyl)-CA-PEG_8_]-5-propargylamido-dUTP (**22**) 

Compound **13** (138 mg, 200 µmol) was dissolved in anhydrous DMF (1.5 mL) and TSTU (60 mg, 200 µmol) and DIPEA (70 µL, 400 µmol) added in quick succession. After 2 h. stirring at r.t., 5-(3-aminoprop-1-ynyl)-2′-deoxyuridine-5′-triphosphate (**9**, 100 µmol) in sodium borate buffer (1.5 mL of a 0.1 M solution) was added and stirring continued for 24 h. The crude reaction mixture was analysed by analytical RP-HPLC (10% B for 5 min then 10–100% B over 30 min; (A = 100 mM TEAB, B = 100 mM TEAB/50% MeCN), and the product (retention time 26 min.) was used without further purification. Aq. NaOH (4 mL of a 1 M solution) was then added and after stirring at r.t. for 2 h., the mixture was neutralised by dropwise addition of aqueous acetic acid (1 M). The crude reaction mixture was analysed by analytical RP-HPLC (5% B for 5 min then 5–100% B over 30 min (A = 100 mM TEAB, B = 100 mM TEAB/50% MeCN) which showed the product at 16.5 min retention time. Subsequent purification by preparative RP-HPLC using the same gradient gave the product 23 (61 µmol, 60%). This was converted to the corresponding Na salt using Dowex ion exchange resin (Na^+^ form). ^31^P NMR (101 MHz, D_2_O): δ_P_ −6.67 (d, *J* = 20.8 Hz), −11.40 (d, *J* = 19.2 Hz), −22.60 (t, *J* = 20.3 Hz) ppm; ES-MS *m*/*z* 1137 [M + H]^+^.

#### 3.2.10. [(3,5-Dicarboxy-1-benzenecarbonyl)-CA-PEG_16_]-5-propargylamido-dUTP (**23**)

Compound **14** (200 µmol) was dissolved in anhydrous DMF (2 mL) and TSTU (60 mg, 200 µmol) and DIPEA (70 µL, 400 µmol) added in quick succession. After 2 h. stirring at r.t., 5-(3-aminoprop-1-ynyl)-2′-deoxyuridine-5′-triphosphate (**9**) (100 µmol) in sodium borate buffer (2 mL of a 0.1 M solution) was added and the mixture stirred for 24 h. The crude reaction mixture was analysed by analytical RP-HPLC (10% B for 5 min then 10–100% B over 30 min; (A = 100 mM TEAB, B = 100 mM TEAB/50% MeCN) and the product (retention time 30 min.) was used without further purification. Aq NaOH (3.2 mL of a 1 M solution) was then added and the mixture stirred for 2 h. at r.t. then neutralised by dropwise addition of acetic acid (1 M). The crude reaction mixture was analysed by analytical RP-HPLC (5% B for 5 min then 5–100% B over 30 min; (A = 100 mM TEAB, B = 100 mM TEAB/50% MeCN) which showed the product at 18 min retention time. Subsequent purification by preparative RP-HPLC using the same gradient gave the product **24** (59 µmol, 59%). This was converted to the corresponding Na salt using Dowex ion exchange resin (Na^+^ form). ^31^P NMR (101 MHz, D_2_O) δ_H_ −6.34 (d, *J* = 20.4 Hz), −11.30 (d, *J* = 19.9 Hz), −22.58 (t, *J* = 19.4 Hz) ppm; ES-MS *m*/*z* 779 [M − 2H]^2^^−^.

#### 3.2.11. [3,5-Dicarboxy-1-benzenecarbonyl)-CA-PEG_24_]-5-propargylamido-dUTP (**24**) 

Compound **17** (88 mg, 63 µmol) was dissolved in anhydrous DMF (1 mL) and TSTU (19 mg, 63 µmol,) and DIPEA (22 µL, 126 µmol) added in quick succession. After 2 h. stirring at r.t., 5-(3-aminoprop-1-ynyl)-2′-deoxyuridine-5′-triphosphate (**9**, 31.6 µmol) in sodium borate buffer (1 mL of a 0.1 M solution) was added and the mixture stirred for 24 h. The crude reaction mixture was analysed by analytical RP-HPLC (10% B for 5 min then 10–100% B over 30 min; (A = 100 mM TEAB, B = 100 mM TEAB/50% MeCN) and the product (retention time 32 min) was used without further purification. Aq NaOH (4 mL of a 1 M solution) was then added and the mixture stirred for 2 h at r.t., then neutralised by dropwise addition of acetic acid (1 M). The crude reaction mixture was analysed by analytical RP-HPLC (5% B for 5 min then 5–100% B over 30 min; (A = 100 mM TEAB, B = 100 mM TEAB / 50% MeCN), which showed the product at 18 min retention time. Subsequent purification by preparative RP-HPLC using the same gradient gave the product (60 µmol, 60%). This was converted to the corresponding Na salt using Dowex ion exchange resin (Na^+^ form). ^31^P NMR (101 MHz, D_2_O) δ_P_ −7.70 (d, *J* = 16.5 Hz), −11.48 (d, *J* = 18.4 Hz), −22.78 (t, *J* = 19.2 Hz) ppm.

#### 3.2.12. [3,5-Dicarboxy-1-benzenecarbonyl)-CA-PEG_8_]-5-propargylamido-dCTP (**25**) 

TSTU (90 mg, 300 μmol) and DIPEA (105 μL, 600 μmol) were added successively to a solution of compound **13** (207 mg, 300 μmol) in anhydrous DMF (2.25 mL). After stirring for 2 h at r.t., a solution of 5-(3-aminoprop-1-ynyl)-2′-deoxycytidine-5′-triphosphate (**10**, 143 μmol) in sodium borate buffer (0.1 M, pH 8.5, 2.25 mL) was added and stirring continued for 24 h. The mixture was then evaporated. Purification by prep-RP-HPLC (5–100% B over 30 min, where A = 0.1 M TEAB and B = 50% MeCN/0.1 M TEAB) gave the desired product (82 μmol, 57%) that eluted with a retention time of retention time of 22.2 min. After characterisation by ES-MS (Acc. Mass [ESI-]: Calculated for C_44_H_69_N_5_O_27_P_3_ [M − H]^−^: 1192.3393 Observed: 1192.3375 [M − H] the purified nucleotide was dissolved in water (1.5 mL), aq. sodium hydroxide (2 M, 1.5 mL) added and the solution stirred at r.t. for 2 h. The mixture was then neutralised by dropwise addition of aq. acetic acid (1 M). Analysis of the reaction by RP-HPLC (5–95% B over 45 min, where A = 0.1 M TEAB and B = 50% MeCN/0.1 M TEAB) showed the product with a retention time of 13 min. Purification by prep-RP-HPLC (gradient as above) gave the triphosphate **25** (67 μmol, 82%). ^31^P NMR (101 MHz, D2O) δ_P_ −8.25 (d, *J* = 20.0 Hz), −11.52 (d, *J* = 20.3 Hz), −22.57 (t, *J* = 19.9 Hz); HR ES-MS *m*/*z* calcd for C_40_H_61_N_5_O_27_P_3_ [M − H]^−^ 1136.2767, found 1136.2736.

#### 3.2.13. [3,5-Dicarboxy-1-benzenecarbonyl)-CA-PEG_4_]-5-propargylamido-dCTP (**26**) 

TSTU (82 mg, 272 μmol) and DIPEA (95 μL, 544 μmol) were added successively to a stirred solution of compound **22** (136 mg, 272 μmol) at r.t. in anhydrous DMF (2.25 mL) under N_2_. After 2 h, a solution of 5-(3-aminoprop-1-ynyl)-2′-deoxycytidine-5′-triphosph ate (**10**, 129 μmol) in sodium borate buffer (0.1 M, pH 8.5, 2.25 mL) was added and stirring continued for 24 h. The mixture was then evaporated. Purification by prep-RP-HPLC (5–65% B over 30 min where A = 0.1 M TEAB and B = 50% MeCN/0.1 M TEAB) gave the desired product (96 μmol, 74%) that eluted with a retention time of retention time 26.7 min. After characterisation by ES-MS (*m*/*z* 1002 [M + H]^+^) the purified nucleotide was dissolved in water (1.5 mL), aq. sodium hydroxide (2 M, 1.5 mL) added and the solution stirred at r.t. for 2h. The mixture was then neutralised by dropwise addition of aq. acetic acid (1 M). Analysis of the reaction by RP-HPLC (5–65% B over 30 min, where A = 0.1 M TEAB and B = 50% MeCN/0.1 M TEAB) showed the product with a retention time of 10.3 min. Purification by prep-RP-HPLC (gradient as above) gave the title triphosphate **26** (79 μmol, 61%). ^1^H-NMR (250 MHz, D_2_O) ^31^P NMR (101 MHz, D_2_O) δ_P_ −6.58 (d, *J* = 21.1 Hz), −11.42 (d, *J* = 19.9 Hz), −22.72 (t, *J* = 20.6 Hz) ppm; HR ES-MS *m*/*z* calcd for C_31_H_41_N_5_O_23_P_3_ [M − H]^−^ 944.1442, found 944.1405.

#### 3.2.14. [3,5-Dicarboxy-1-benzenecarbonyl)-CA-PEG_6_]-5-propargylamido-dUTP) (**27**) 

Diethyl 1,3,5-benzenetricarboxylate (**15**, 5 mg, 18 µmol) was dissolved in anhydrous DMF (1.25 mL) and TSTU (6 mg, 18 µmol) and DIPEA (60 µL, 36 µmol) added in quick succession. After 2 h. stirring at r.t., PEG_6_-propargyl-dUTP (**33**, 8.3 µmol) in sodium borate buffer (1.25 mL of a 0.1 M solution) was added and stirring continued for 20 h. The crude reaction mixture was analysed by analytical RP-HPLC (10% B for 5 min; ramp to 100% B over 30 min; hold 5 min (A = 100 mM TEAB, B = 100 mM TEAB/50% MeCN), UV detection at 288 nm) which showed the product at 25 min retention time. Aq. 1M NaOH solution (2 mL) was added and stirring continued for 2 h at r.t. After this time the reaction mixture was neutralised by dropwise addition of aq acetic acid (1 M). The crude reaction mixture was analysed by analytical RP-HPLC (10% B for 5 min then 10–100% B over 35 min; (A = 100 mM TEAB, B = 100 mM TEAB/50% MeCN), which showed the product at 19.5 min retention time. Subsequent purification by preparative RP-HPLC using the same gradient gave the product **28** (3.90 µmol, 47%). ^31^P-NMR (101 MHz, D_2_O) δ_P_ −6.92 (d, *J* = 18.5 Hz), −11.45 (d, *J* = 19.5 Hz), −22.67 (t, *J* = 18.6 Hz) ppm; HR ES-MS *m*/*z* calcd for C_41_H_53_N_3_O_26_P_3_ [M − H]^−^ 1096.2130, found 1096.2080.

#### 3.2.15. 3,6,9,12,15,18-Hexaoxaheneicos-20-yn-1-ol (**28**) 

Hexaethylene glycol (5 g, 17.7 mmol) and NaH (60% in oil,1.06 g, 26.6 mmol) were dissolved in anhydrous DMF (100 mL) at 0 °C and stirred for 2 h at r.t. Propargyl bromide (2.96 mL, 26.6 mmol) was added dropwise and the reaction mixture stirred for 16 h at r.t. MeOH (10 mL) was then added and the reaction mixture evaporated. Purification by silica column chromatography (0–10% MeOH in EtOAc) gave a yellow oil (1.73 g, 31%). *R*_f_ 0.6 (10% MeOH in EtOAc); ^1^H-NMR δ (250 MHz, CDCl_3_) δ_H_ 4.22 (d, *J* = 2.5 Hz 2H, OCH_2_C≡CH), 3.73–3.64 (m, 24H, 6 × OCH_2_CH_2_), 2.45 (s, 1H, C≡CH); ^13^C-NMR δ (101 MHz, CDCl_3_) δ_C_ 72.6, 70.6, 70.4, 70.3, 69.1, 61.8, 58.4 ppm; HR ES-MS *m*/*z* calcd for C_15_H_28_O_7_Na [M + Na]^+^ 343.1733, found 343.1719.

#### 3.2.16. 1-(4-Bromomethylbenzyl)-1-(1,3,6,9,12,15,18-hexaoxaheneicos-20-yne (**29**) 

Compound **28** (1.73 g, 5.34 mmol) was dissolved in anhydrous DMF (100 mL) at 0 °C and NaH (60% in oil, 259 mg, 6.48 mmol) was added. After stirring for 2 h, 1,4-bis(bromomethyl)benzene (1.71 g, 6.48 mmol) was added gradually. The reaction was stirred for 16 h at r.t., then the solution was evaporated. Purification by silica column chromatography (0–1% MeOH in EtOAc) gave the product as an oil (1.13 g, 42%). *R*_f_ 0.36 (EtOAc); ^1^H-NMR (400 MHz, CDCl_3_) δ_H_ 7.43–7.32 (m, 4H, phenyl CH), 4.57 (s, 2H, CH_2_), 4.51 (s, 2H, CH_2_), 4.22 (d, *J* = 2.4 Hz, 2H, OCH_2_C≡CH), 3.78–3.59 (m, 24H, 6 × OCH_2_CH_2_), 2.45 (t, *J* = 2.4 Hz, 1H, C≡CH) ppm; ^13^C-NMR (101 MHz, CDCl_3_) δ_C_ 138.7, 137.1, 129.1, 128.1, 74.5, 72.8, 70.6, 70.4, 69.6, 69.1, 58.4, 33.4 ppm; HR ES-MS *m*/*z* calcd for C_23_H_36_O_7_Br [M + H]^+^ 503.1644, found 503.1627.

#### 3.2.17. 1-(4-Phthalimidomethylbenzyl)-1-(1,3,6,9,12,15,18-hexaoxaheneicos-20-yne (**30**)

Compound **29** (1.31 g, 2.25 mmol) and potassium phthalimide (458 mg, 2.48 mmol, 1.1 eq.) were dissolved in anhydrous DMF (60 mL) and heated to reflux for 16 h. After cooling to r.t., the solvent was evaporated and the residue dissolved in EtOAc (50 mL) and washed with saturated brine. The aqueous layer was extracted with EtOAc (3 × 40 mL) and the organic layers were dried (Na_2_SO_4_) and concentrated to give a thick brown oil, which was purified by silica column chromatography (EtOAc) to give the desired product (1.10 g, 86%). TLC: *R*_f_ 0.38 (EtOAc); ^1^H-NMR (400 MHz, CDCl_3_) δ_H_ 7.88–7.85 (m, 2H, phenyl CH (phthalimide)), 7.74–7.72 (m, 2H, phenyl CH (phthalimide)), 7.44 (m, 2H, phenyl CH), 7.42 (m, 2H, phenyl CH), 4.86 (s, 2H, CH_2_), 4.54 (s, 2H, CH_2_), 4.23 (d, *J* = 2.4 Hz, 2H, OCH_2_C≡CH), 3.71–3.65 (m, 24H, 6 × OCH_2_CH_2_), 2.45 (t, *J* = 2.4 Hz, 1H, C≡CH) ppm; ^13^C-NMR δ_C_ (101 MHz, CDCl_3_): 134.0, 128.7, 128.1, 123.4, 74.6, 72.9, 70.6, 70.4, 69.4, 69.1, 58.4, 41.4 ppm; HR ES-MS *m*/*z* calcd for C_31_H_39_NO_9_Na [M + Na]^+^ 592.2523, found 592.2544.

#### 3.2.18. 5-[(4-Phthalimidomethylbenzyl)-PEG_6_-propargyl)]-3′-O-acetyl-2′-deoxyuridine (**32**)

5-Iodo-3′-*O*-acetyl-2′-deoxyuridine (**31**, 250 mg, 632 µmol) [27], copper (I) iodide (24 mg, 126 µmol), Pd (PPh_3_)_4_ (73 mg, 63 µmol) and triethylamine (176 µL, 1.26 mmol) were dissolved in anhydrous DMF (5 mL). Compound **31** (900 mg, 1.58 mmol) dissolved in anhydrous DMF (5 mL) and then added portionwise to the mixture over 30 min. After stirring for 18 h, a 5% aq. solution of disodium EDTA (12 mL) was added and the mixture stirred until a cloudy green precipitate formed. The solvent was then evaporated and the residue dissolved in EtOAc. The EtOAc solution was then washed with water (3 × 30 mL), dried (Na_2_SO_4_) and evaporated. The crude product was purified by silica column chromatography (0–5% MeOH in DCM) to give a white solid (201 mg, 38%). *R*_f_ 0.22 (5% MeOH in DCM); ^1^H-NMR δ (400 MHz, CDCl_3_) δ_H_ 9.00 (br s, 1H, NH), 8.38 (s, 1H, H6), 7.95–7.75 (m, 2H, phenyl CH (phthalimide)), 7.78–7.62 (m, 2H, phenyl CH (phthalimide)), 7.40 (d, *J* = 8.1 Hz, 2H, phenyl CH), 7.31 (d, *J* = 2.2 Hz, 2H, phenyl CH), 6.33 (dd, *J* = 8.3, 5.8 Hz, 1H, H1′), 5.35 (m, 1H, H4′), 4.84 (s, 2H, CH_2_), 4.52 (s, 2H, CH_2_), 4.37 (s, 2H, OCH_2_C≡CH), 4.13 (d, *J* = 1.7 Hz, 1H, H3′), 3.96–3.84 (m, 2H, 5′CH_2_), 3.79–3.50 (m, 24H, 6 × OCH_2_CH_2_), 2.52–2.25 (m. 2H, 2′CH_2_), 2.10 (s, 3H, COOCH_3_); ^13^C-NMR (101 MHz, CDCl_3_) δ_C_ 149.1, 144.2, 134.0, 132.1, 128.7, 128.1, 127.9, 123.4, 85.8, 85.7, 75.4, 72.8, 70.5, 70.4, 69.4, 69.1, 62.3, 59.2, 41.3, 38.3, 21.0, 1.0 ppm; Mass Spec: ES+ *m*/*z* 838.3 [M + H]^+^.

#### 3.2.19. 5-[(4-Aminomethylbenzyl)-PEG_6_-propargyl)]-dUTP (**33**) 

5-(Phthalimido-PEG_6_-propargyl)-3′-O-acetyl-2′-deoxyuridine (**32**, 100 mg, 119 µmol) was dried in a vacuum dessicator over P_2_O_5_ overnight. The nucleoside was then dissolved in 1:3 *v*/*v* dioxane/pyridine (480 µL) in the reaction flask before salicyl chlorophosphite (286 µL of a 1 M solution in dioxane, 286 µmol) was added. After 30 min bis(tri-*n*-butylammonium) pyrophosphate (358 µL of a 0.5 M solution in DMF, 179 µmol) in tributylamine (86 µL, 358 µmol) was added in one portion to the reaction flask. After stirring for a further 10 min. iodine (1.2 mL of a 1% solution in 50/50 pyridine/H_2_O) was added, and after another 15 min stirring NaHSO_3_ (0.5 mL of a 5% aqueous solution) was added. The reaction mixture was then evaporated to a gum before addition of methylamine (10 mL of a 10% aqueous solution). After 18 h stirring, the methylamine was removed under reduced pressure. The crude product was purified by MPLC ion-exchange chromatography (gradient 50 mM–900 mM TEAB, flow rate 8 mL/min, UV detection at 288 nm). Analytical RP-HPLC (10% B for 5 min; ramp to 65% B over 30 min; to 100% B over 1 min; hold 6 min (A = 100 mM TEAB, B = 100 mM TEAB/50% MeCN), UV detection at 288 nm) of the product peak from MPLC ion-exchange chromatography showed that the triphosphate (retention time 27 min). Purification by preparative RP- HPLC using the same gradient gave the product (42 µmol, 35%). ^31^P-NMR (101 MHz, D_2_O) δ_P_ −9.15 (d, *J* = 19.1 Hz), −11.35 (d, *J* = 19.8 Hz), −22.81 (t, *J* = 19.3 Hz) ppm, HR ES-MS *m*/*z* calcd for C_32_H_49_N_3_O_21_P_3_ [M − H]^−^ 904.2071, found 904.2032.

### 3.3. Nucleotide Incorporation Assays

Using either ploymerases HotStarTaq (heated to 95 °C for 3 min prior to use), Phusion, Vent (*exo-*), Klenow (*exo-*), Bst 2.0, Therminator (New England Biolabs, Ipswich, MA, USA), or Omni-KlenTaq DNA polymerase (DNA Technology, St Louis, MO, USA), primer extension reactions were performed as 20 µL reactions comprising of: 1 U polymerase in 1× polymerase specific buffer (provided by DNA Technology, St Louis, MO, USA), 20 nM Cy5-5′-labelled primer and 15 nM template. To investigate the incorporation of a modified dCTP, a nucleotide mix (modified-dCTP, dATP, dGTP, dTTP) was included alongside the three controls (Positive control: dATP, dGTP, dTTP, dCTP; Polymerase fidelity control: dATP, dGTP, dTTP; Negative control: No polymerase). The incorporation of the modified dUTPs were investigated in an analogous manner (modified dUTP replacing dTTP). All dNTP mixes were used at a final concentration of 625 nM. In all cases the polymerase was added lastly to the reaction mixes before the desired incubation period. After incubation (time and temperature indicated in main text/figure legends), each reaction was stopped with the addition of 5 µL complementary sequence (100 µM) in a solution of EDTA (250 mM, pH 8), formamide and Orange G gel loading dye. The reaction mixtures were denatured by heating at 95 °C for 3 min and analysed by electrophoresis on a 17.5% polyacrylamide gel (PAGE). The gel was imaged by fluorescence visualisation facilitated by the use of the Cy5-labelled primer.

## 4. Conclusions

We have described the chemical synthesis and characterisation of four C5-modified dUTP and two C5-modified dCTP analogues that are functionalised with polyanionic side chains linked to the nucleosides with PEG linkers of differing lengths. We have shown that these nucleotides can be incorporated into DNA by a variety of commercially available DNA polymerases, particularly Vent (*exo*-) and Omni Klentaq. Klenow (*exo-*) was less efficient at incorporation of the dCTP analogues compared to the other polymerases tested. Successful incorporation of the modified dNTPs is manifested by a reduced electrophoretic mobility that is dependent of the length of the PEG linker. Although all modified dNTPs were successfully incorporated by all DNA polymerases tested the largest modified dNTP, the PEG_24_-dUTP analogue, hindered the continued extension with both Pfu and Bst 2.0 DNA polymerases. The alkyne-ether linkage also examined showed good incorporation with only Bst 2.0 DNA polymerase failing to continue primer extension after its incorporation. We have therefore demonstrated that these anionically charged PEG modified dNTPs are substrates for a number of DNA polymerases and therefore might be exploited for a variety of biotechnological platforms.

## Data Availability

The data presented in this study are available on request from the corresponding author.

## Supplementary Materials

The following are available online, ^1^H-, ^13^C- and ^31^P-NMR spectra of synthesized compounds.

## Sample Availability

Samples of the compounds where still available can be requested from the corresponding authors.
