# Supplementary material for: Synthesis of Polyanionic C5-Modified 2′-Deoxyuridine and 2′-Deoxycytidine-5′-Triphosphates and Their Properties as Substrates for DNA Polymerases
Source: Molecules. 2021 Apr 13;26(8):2250. doi: 10.3390/molecules26082250 (PMC8069024; doi:10.3390/molecules26082250)

Synthesis of polyanionic C5-modified 2'-deoxyuridine and 2'-deoxycytidine-5'-triphosphates and their properties as substrates for DNA polymerases.

Claire Dutson[1], Esther Allen[1], Mark J. Thompson[1], Joseph H. Hedley[2,†], Heather E. Murton[2,††], David M. Williams[1,*]

[1] *Centre for Chemical Biology, Department of Chemistry, Sheffield Institute for Nucleic Acids, University of Sheffield, S3 7HF, UK.*
[2] *QuantuMDx Group, Lugano Building, 57 Melbourne Street, Newcastle upon Tyne, NE1 2JQ, UK.*
† Present address: *Northumbria University, Sutherland Building, Newcastle-upon-Tyne, NE1 8ST*
†† Present address: Lex Diagnostics, Melbourn Science Park, Melbourn, SG8 6EE.
* Correspondence: d.m.williams@sheffield.ac.uk; Tel.: +44 114 222 9502 (DW)

Supporting information: ^{1}H, ^{13}C and ^{31}P NMR spectra

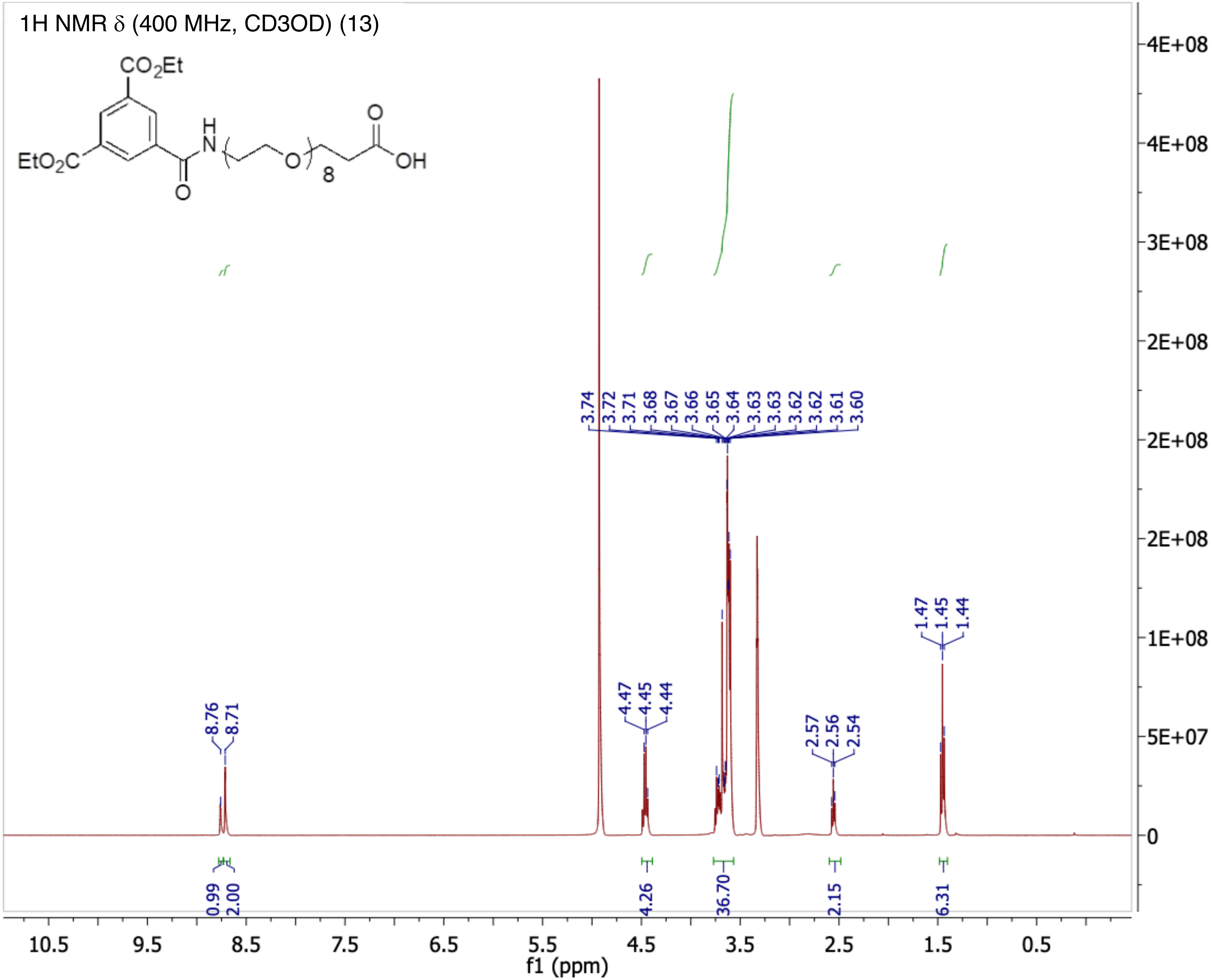
1H NMR δ (400 MHz, CD3OD) (13)
CO2Et
EtO2C
H
N
O
O
8
O
OH
3.74
3.72
3.71
3.68
3.67
3.66
3.65
3.64
3.63
3.63
3.62
3.62
3.61
3.60
8.76
8.71
4.47
4.45
4.44
2.57
2.56
2.54
1.47
1.45
1.44
0.99
2.00
4.26
36.70
2.15
6.31
4E+08
4E+08
3E+08
2E+08
2E+08
2E+08
1E+08
5E+07
0
10.5
9.5
8.5
7.5
6.5
5.5
4.5
3.5
2.5
1.5
0.5
f1 (ppm)

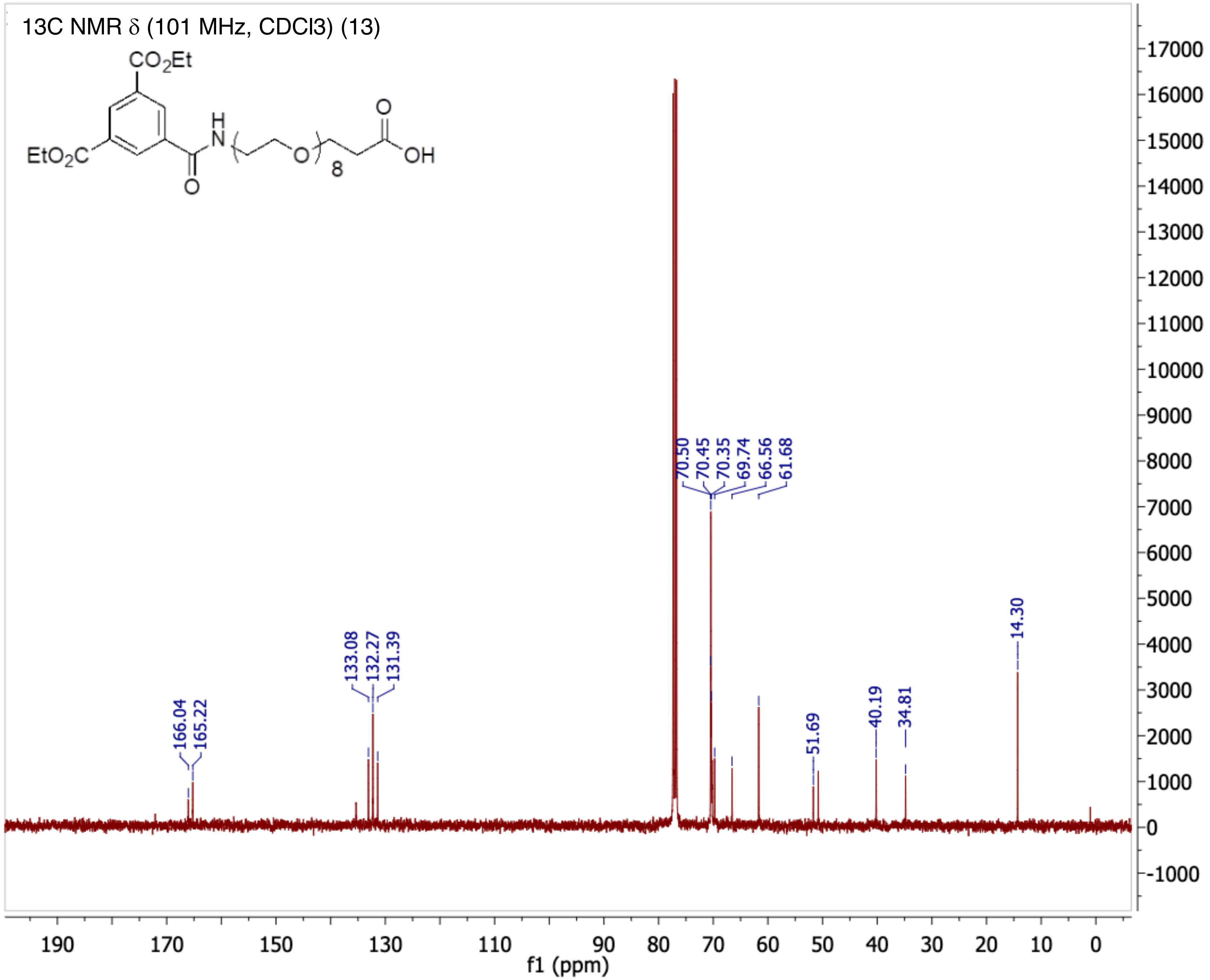
13C NMR δ (101 MHz, CDCl3) (13)
CO2Et
EtO2C
H
N
O
O
8
O
OH
166.04
165.22
133.08
132.27
131.39
70.50
70.45
70.35
69.74
66.56
61.68
51.69
40.19
34.81
14.30
f1 (ppm)

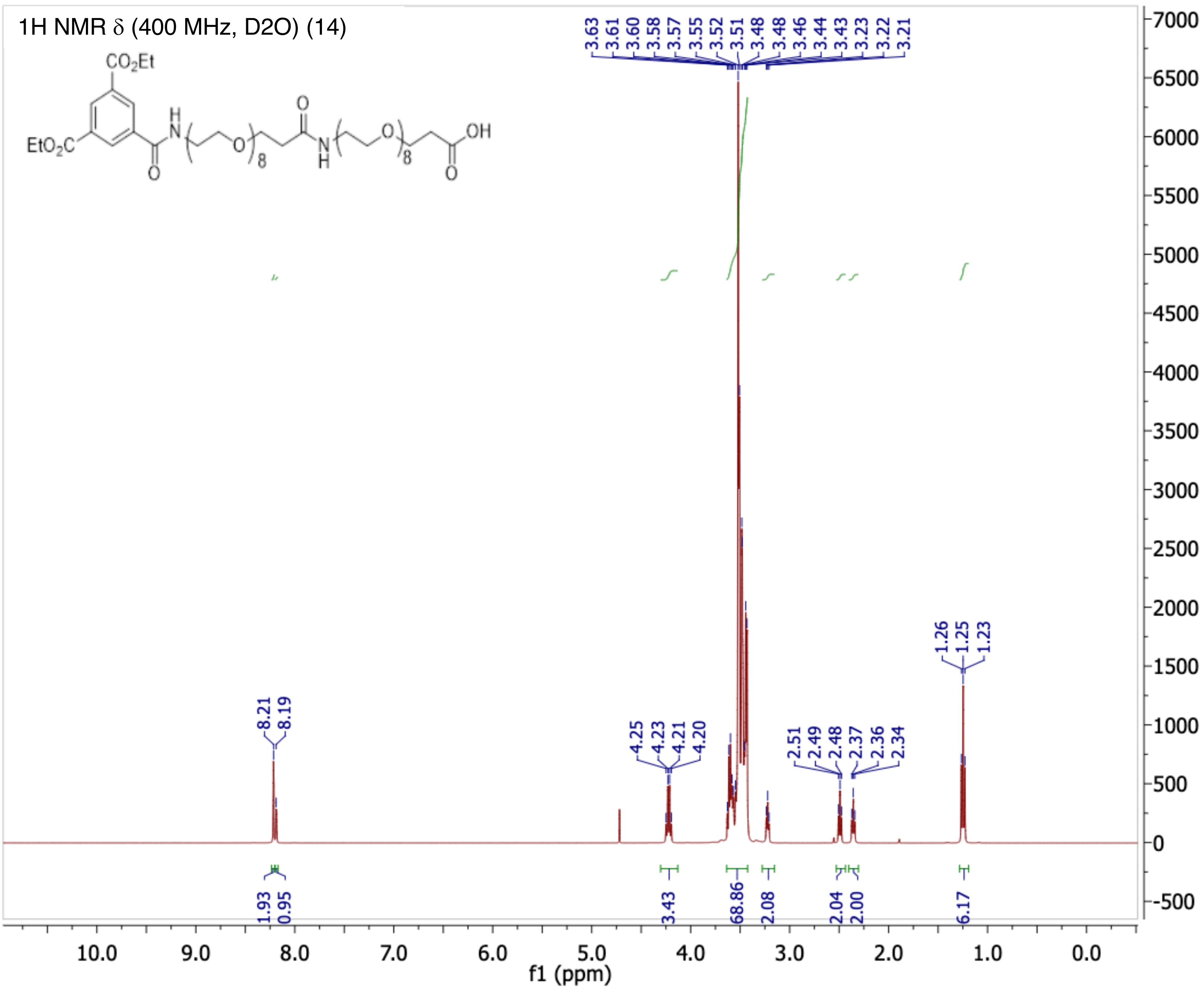
1H NMR δ (400 MHz, D2O) (14)
CO2Et
EtO2C
H
N
O
OH
8
3.63 3.61 3.60 3.58 3.57 3.55 3.52 3.51 3.48 3.48 3.46 3.44 3.43 3.23 3.22 3.21
8.21 8.19
4.25 4.23 4.21 4.20
2.51 2.49 2.48 2.37 2.36 2.34
1.26 1.25 1.23
1.93 0.95 3.43 68.86 2.08 2.04 2.00 6.17
10.0 9.0 8.0 7.0 6.0 5.0 4.0 3.0 2.0 1.0 0.0
f1 (ppm)
7000 6500 6000 5500 5000 4500 4000 3500 3000 2500 2000 1500 1000 500 0 -500

13C NMR δ (101 MHz, D2O) (14)

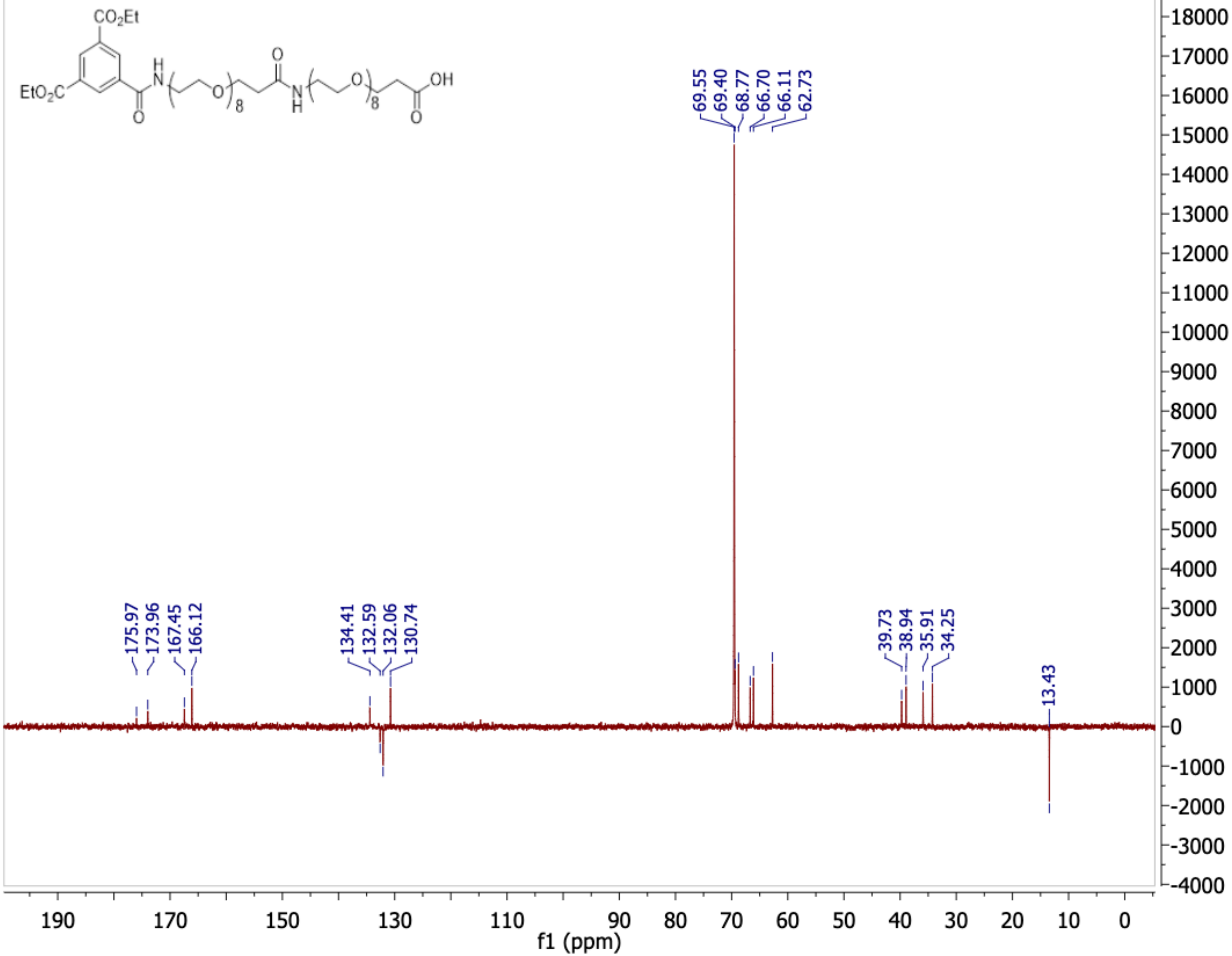

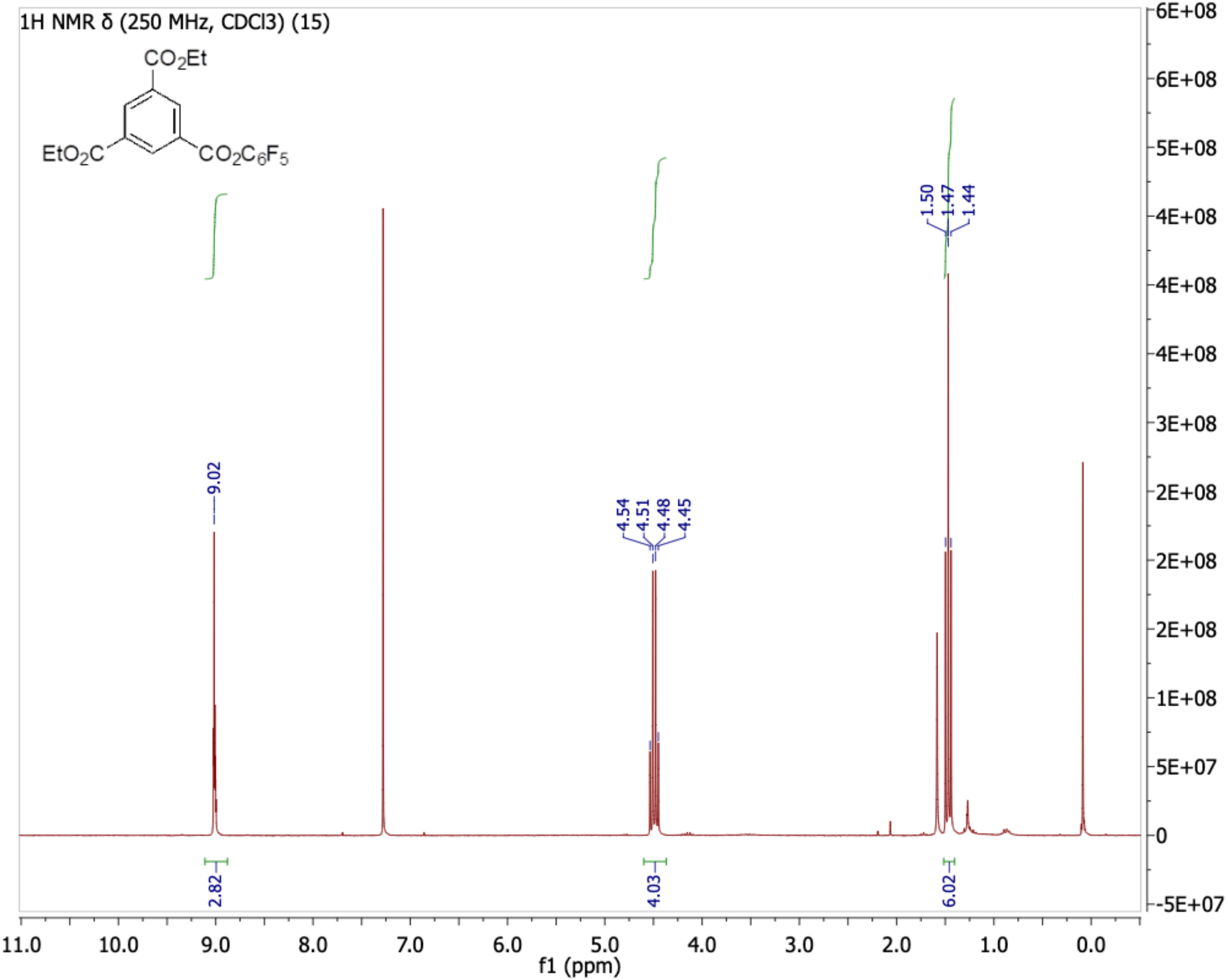

1H NMR δ (250 MHz, CDCl3) (15)
CO_2Et
EtO_2C
$CO_2C_6F_5$
9.02
4.54
4.51
4.48
4.45
1.50
1.47
1.44
2.82
4.03
6.02
6E+08
6E+08
5E+08
4E+08
4E+08
4E+08
3E+08
2E+08
2E+08
2E+08
1E+08
5E+07
0
-5E+07
11.0
10.0
9.0
8.0
7.0
6.0
5.0
4.0
3.0
2.0
1.0
0.0
f1 (ppm)

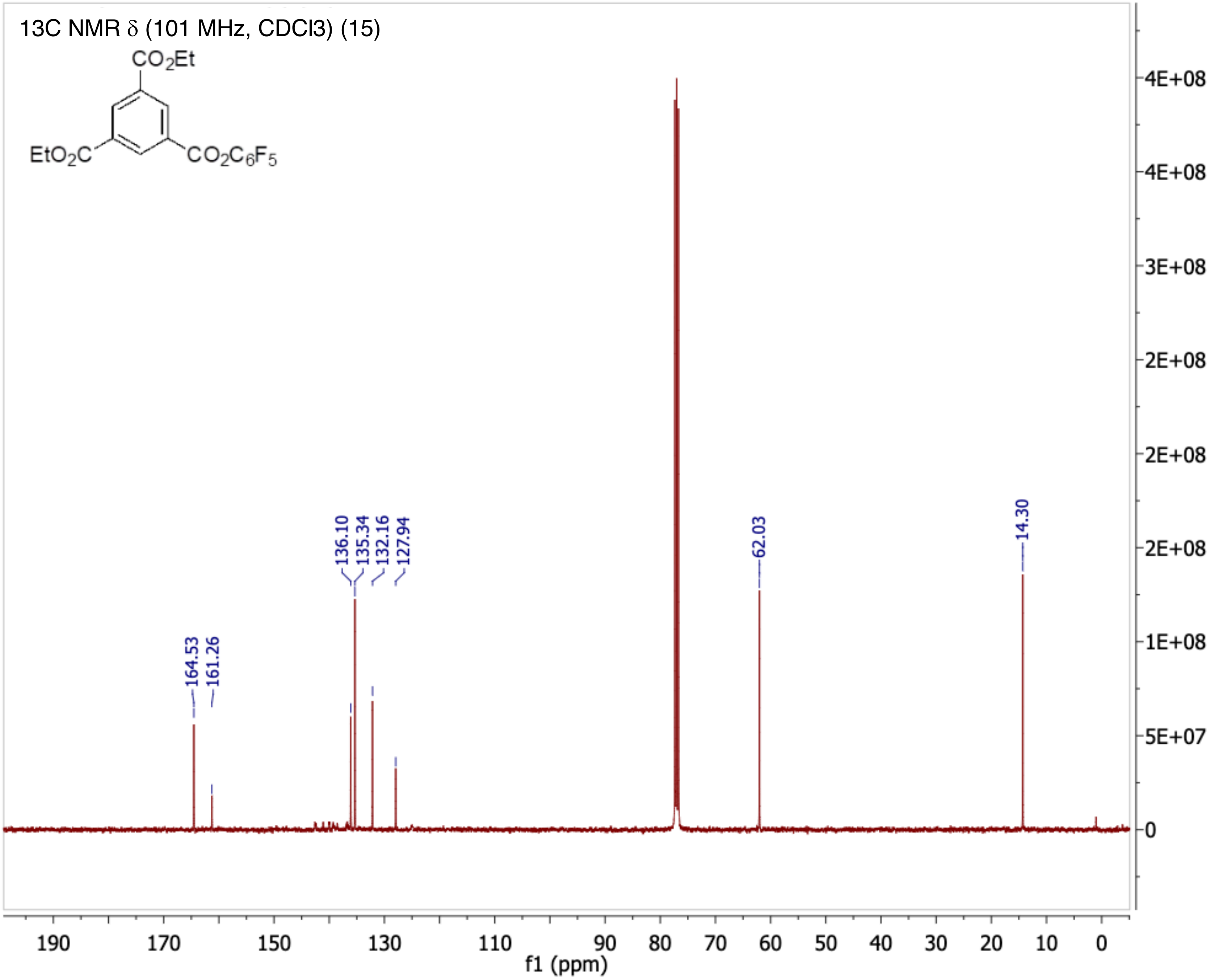

13C NMR δ (101 MHz, CDCl3) (15)
CO2Et
EtO2C
CO2C6F5
164.53
161.26
136.10
135.34
132.16
127.94
62.03
14.30
4E+08
4E+08
3E+08
2E+08
2E+08
2E+08
1E+08
5E+07
0
190
170
150
130
110
90
80
70
60
50
40
30
20
10
0
f1 (ppm)

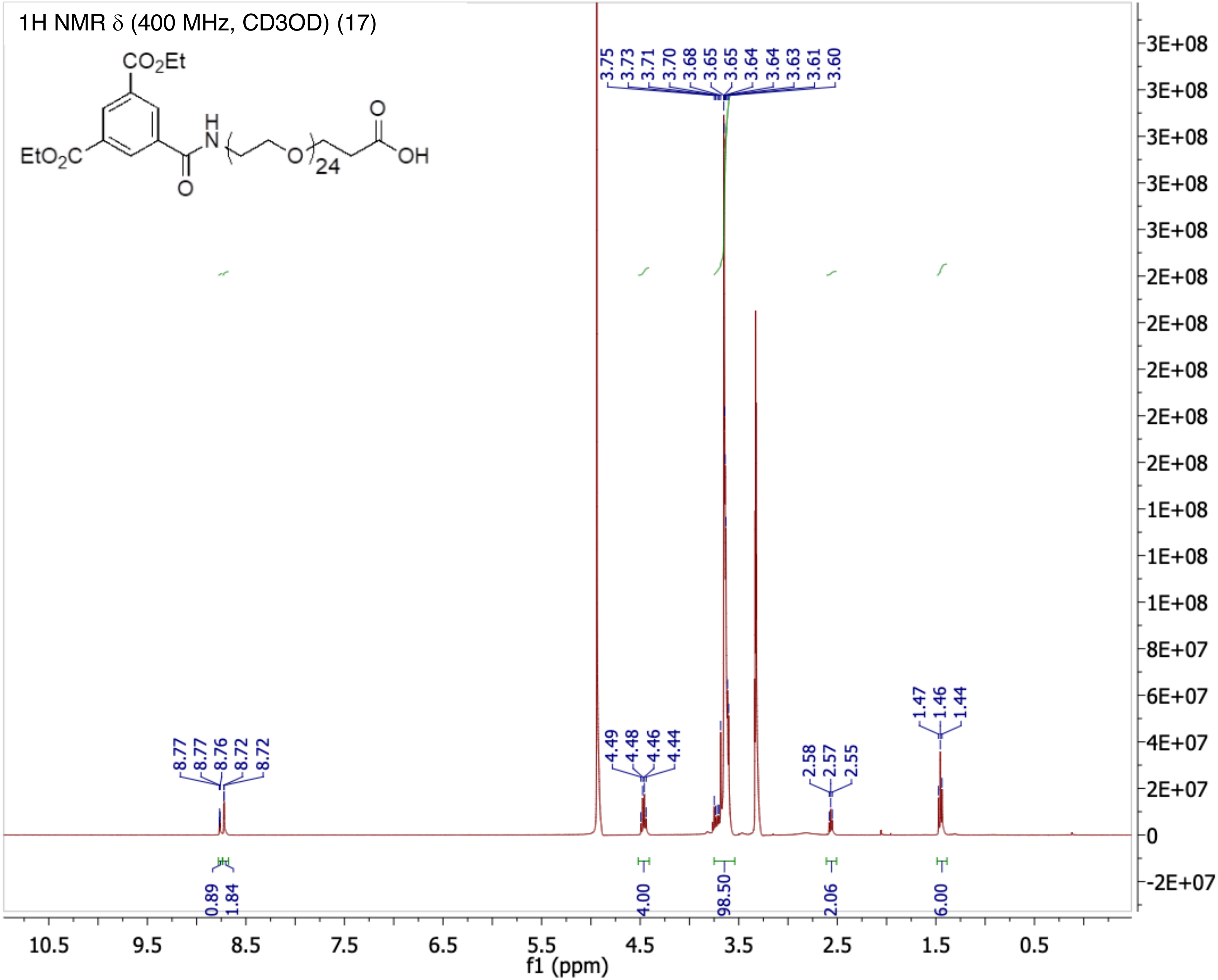
1H NMR δ (400 MHz, CD3OD) (17)
CO2Et
EtO2C
H
N
O
O
24
O
OH
3.75
3.73
3.71
3.70
3.68
3.65
3.65
3.64
3.64
3.63
3.61
3.60
8.77
8.77
8.76
8.72
8.72
4.49
4.48
4.46
4.44
2.58
2.57
2.55
1.47
1.46
1.44
0.89
1.84
4.00
98.50
2.06
6.00
f1 (ppm)

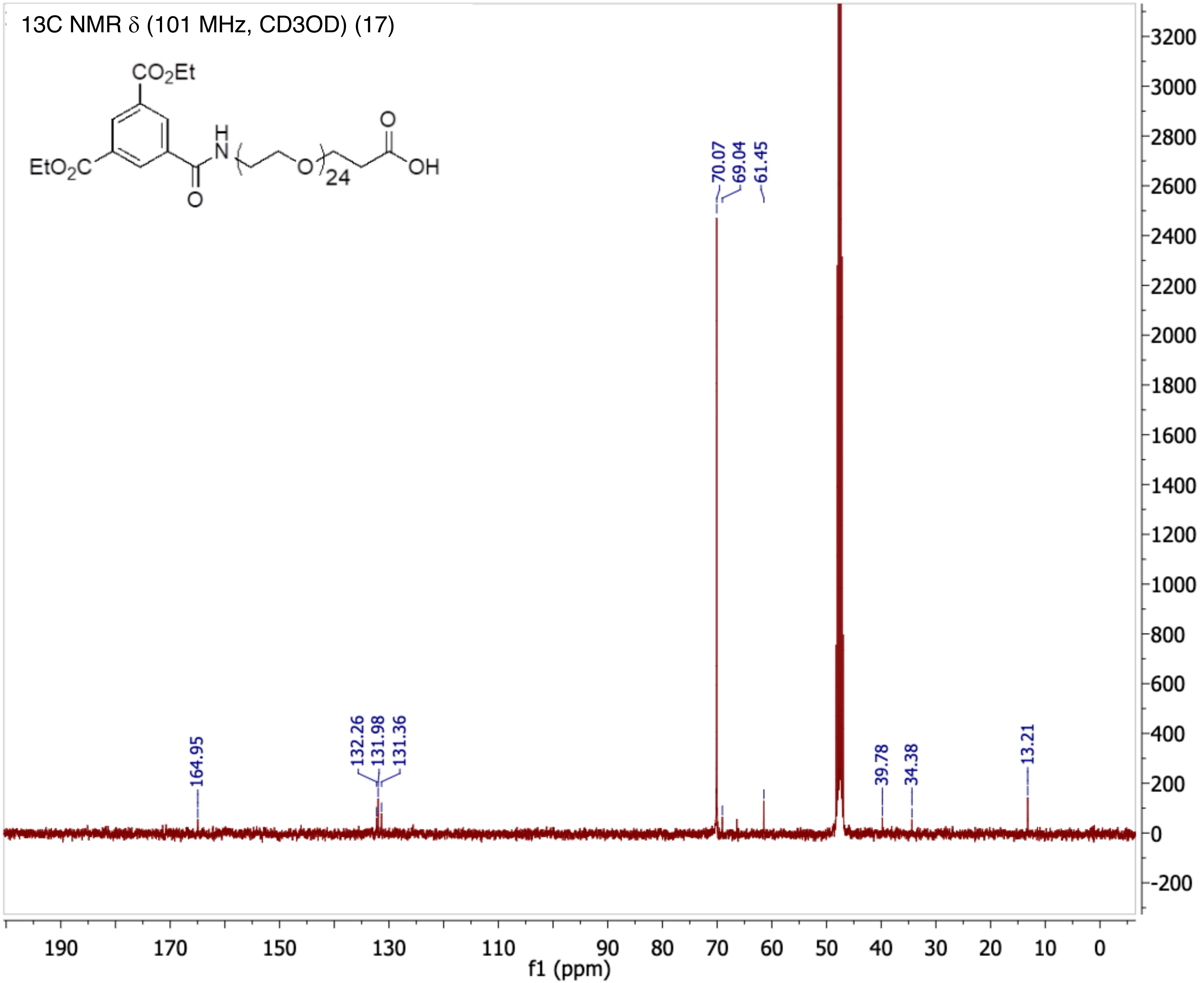
13C NMR δ (101 MHz, CD3OD) (17)
CO2Et
EtO2C
H
N
O
O
24
OH
70.07
69.04
61.45
164.95
132.26
131.98
131.36
39.78
34.38
13.21
f1 (ppm)

1H NMR (250 MHz, CDCl3) (18)

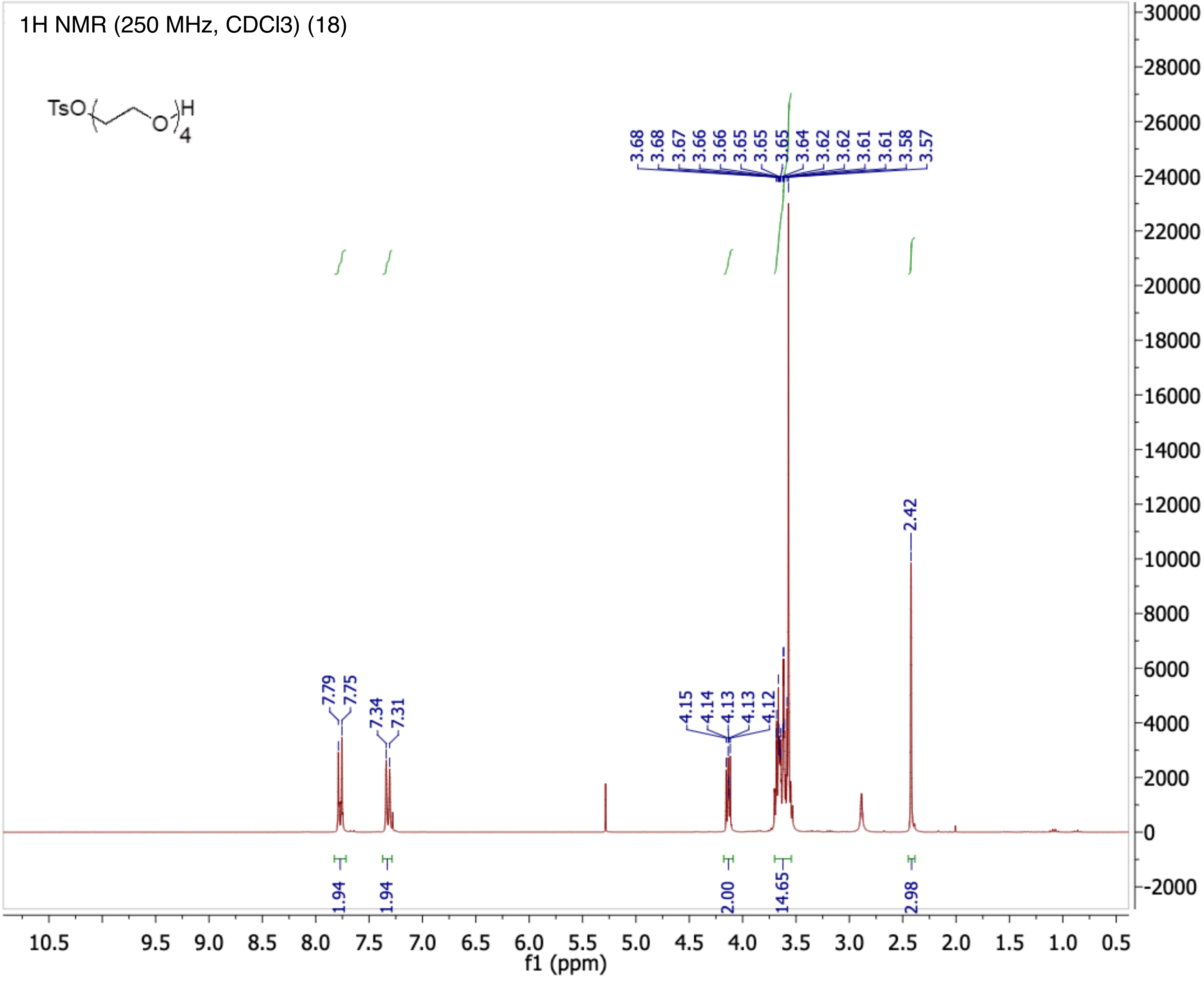

13C NMR (101 MHz, CDCl3) (18)

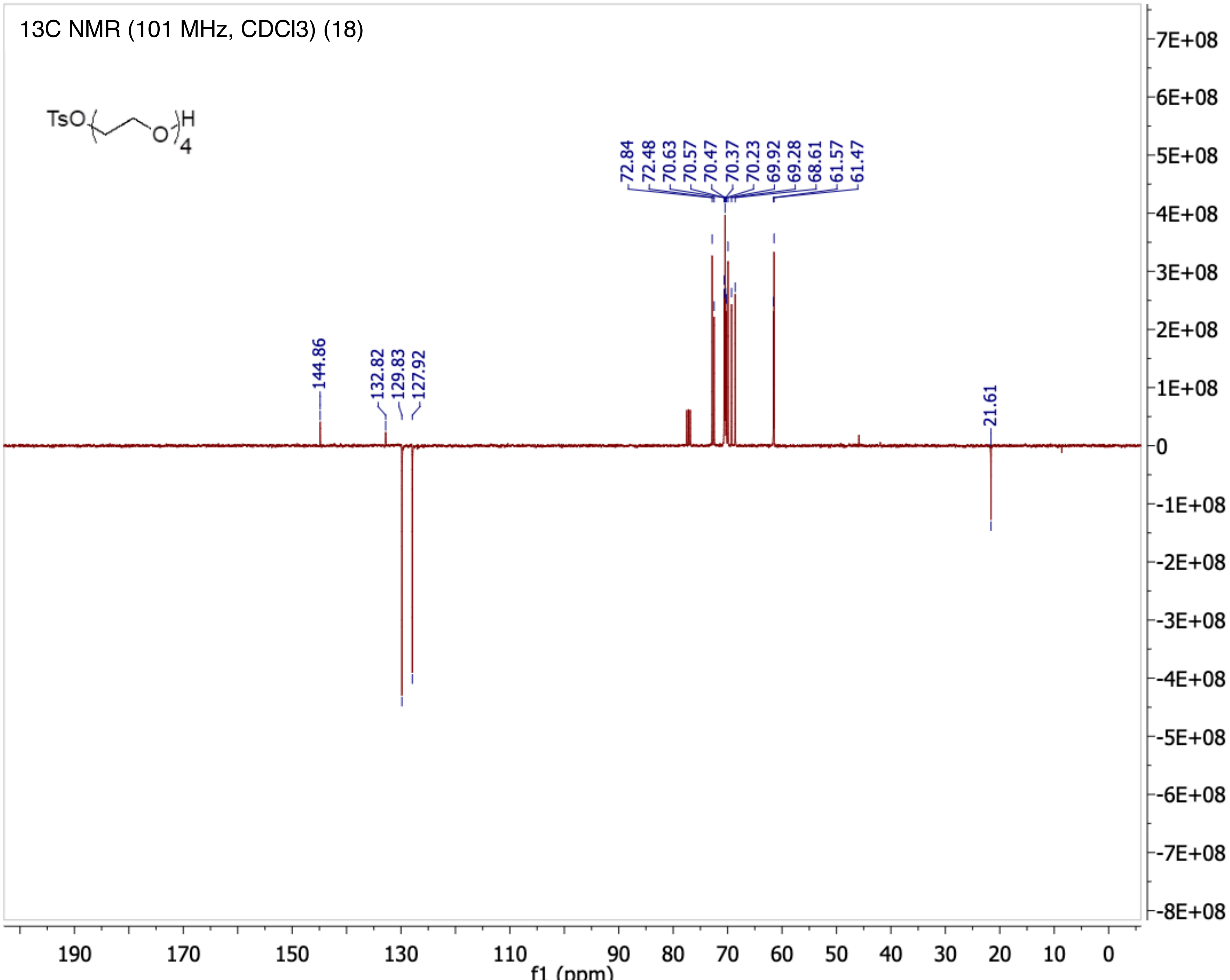

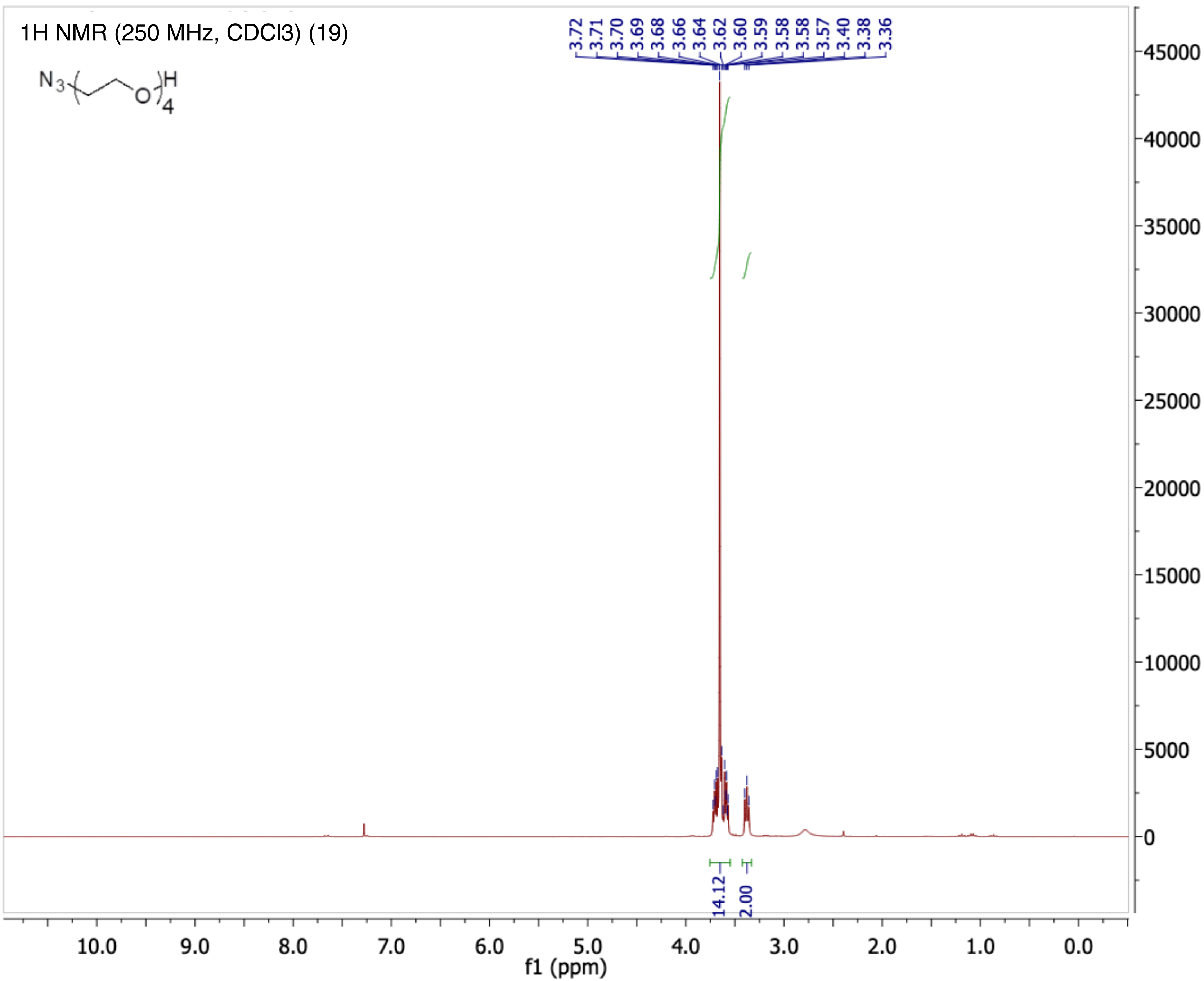
1H NMR (250 MHz, CDCl3) (19)
N3 O H 4
3.72
3.71
3.70
3.69
3.68
3.66
3.64
3.62
3.60
3.59
3.58
3.58
3.57
3.40
3.38
3.36
14.12
2.00
10.0
9.0
8.0
7.0
6.0
5.0
4.0
3.0
2.0
1.0
0.0
f1 (ppm)
45000
40000
35000
30000
25000
20000
15000
10000
5000
0

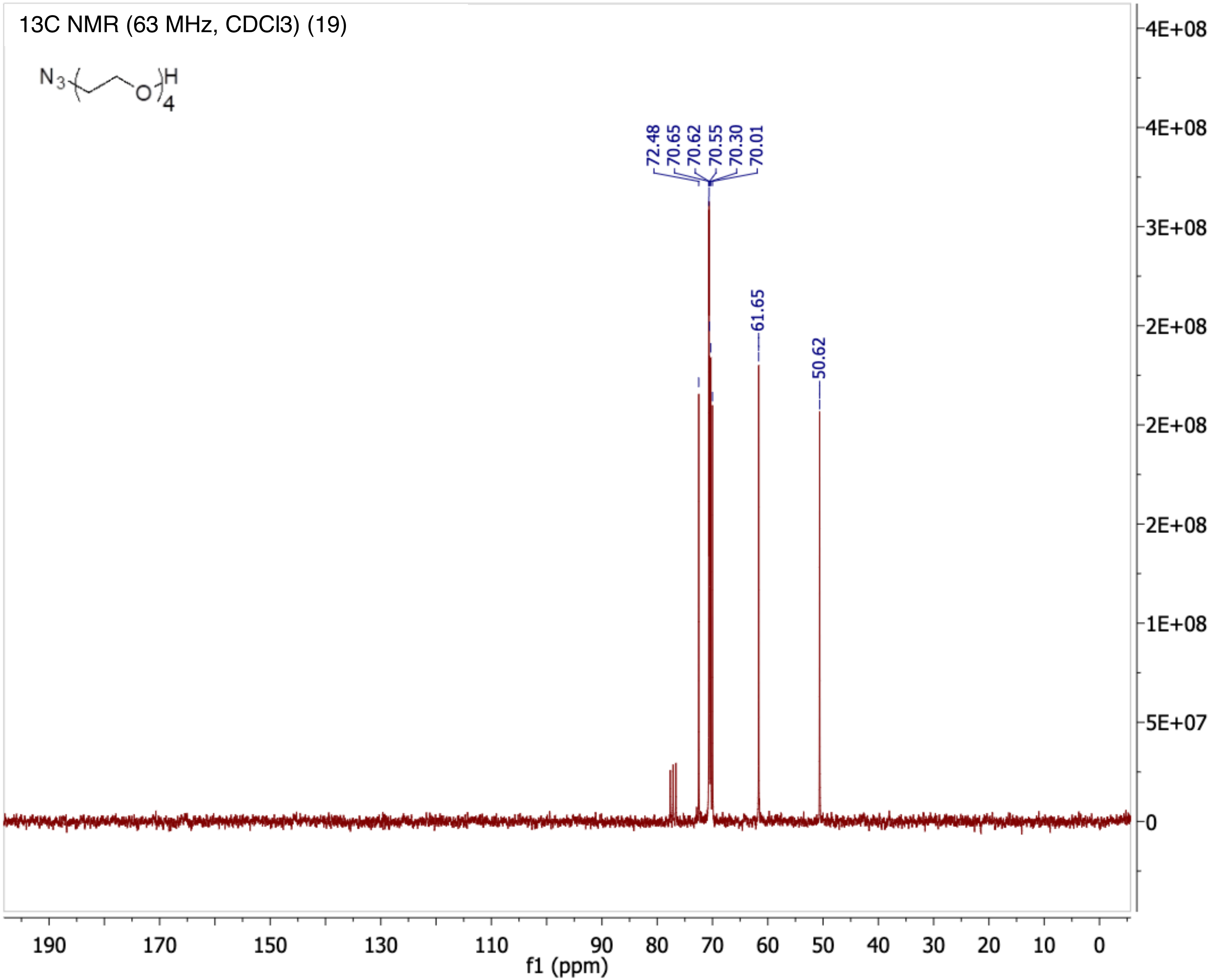
13C NMR (63 MHz, CDCl3) (19)
N3 O H 4
72.48
70.65
70.62
70.55
70.30
70.01
61.65
50.62
4E+08
4E+08
3E+08
2E+08
2E+08
2E+08
1E+08
5E+07
0
190
170
150
130
110
90
80
70
60
50
40
30
20
10
0
f1 (ppm)

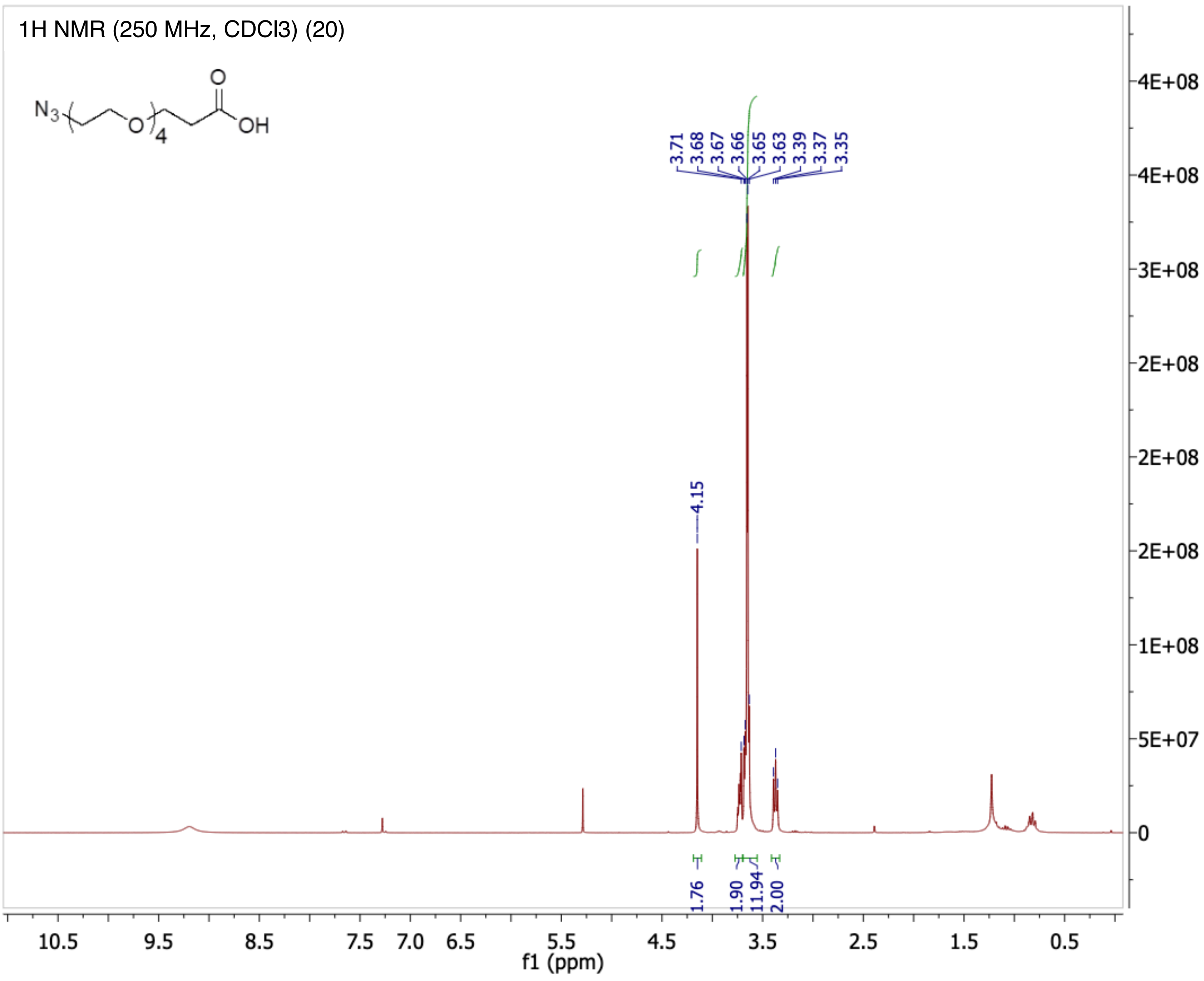
1H NMR (250 MHz, CDCl3) (20)
N3
O
4
O
OH
3.71
3.68
3.67
3.66
3.65
3.63
3.39
3.37
3.35
4.15
1.76
1.90
11.94
2.00
4E+08
4E+08
3E+08
2E+08
2E+08
2E+08
1E+08
5E+07
0
10.5
9.5
8.5
7.5
7.0
6.5
5.5
4.5
3.5
2.5
1.5
0.5
f1 (ppm)

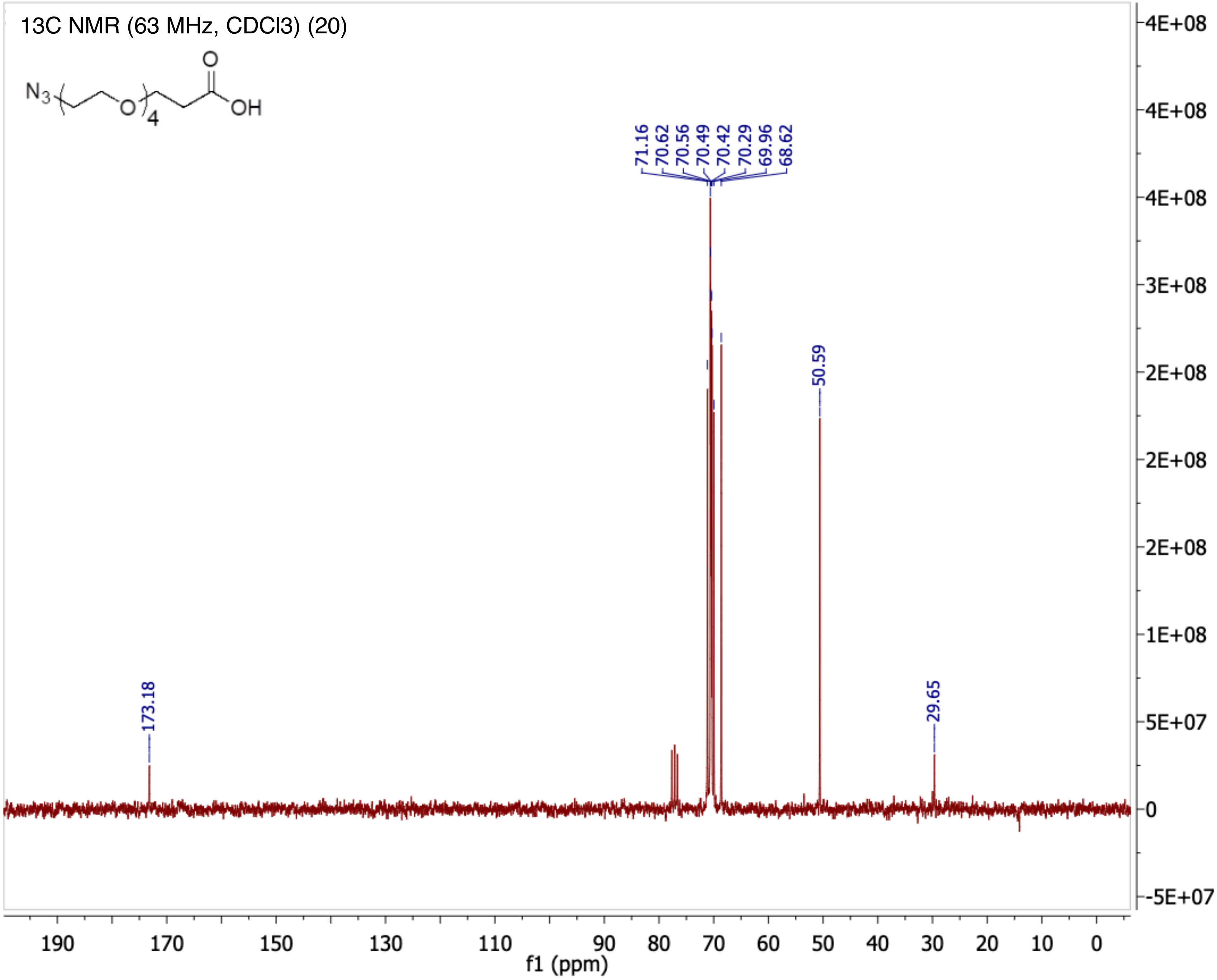
13C NMR (63 MHz, CDCl3) (20)
N3
O
4
O
OH
71.16
70.62
70.56
70.49
70.42
70.29
69.96
68.62
50.59
173.18
29.65
4E+08
4E+08
4E+08
3E+08
2E+08
2E+08
2E+08
1E+08
5E+07
0
-5E+07
190
170
150
130
110
90
80
70
60
50
40
30
20
10
0
f1 (ppm)

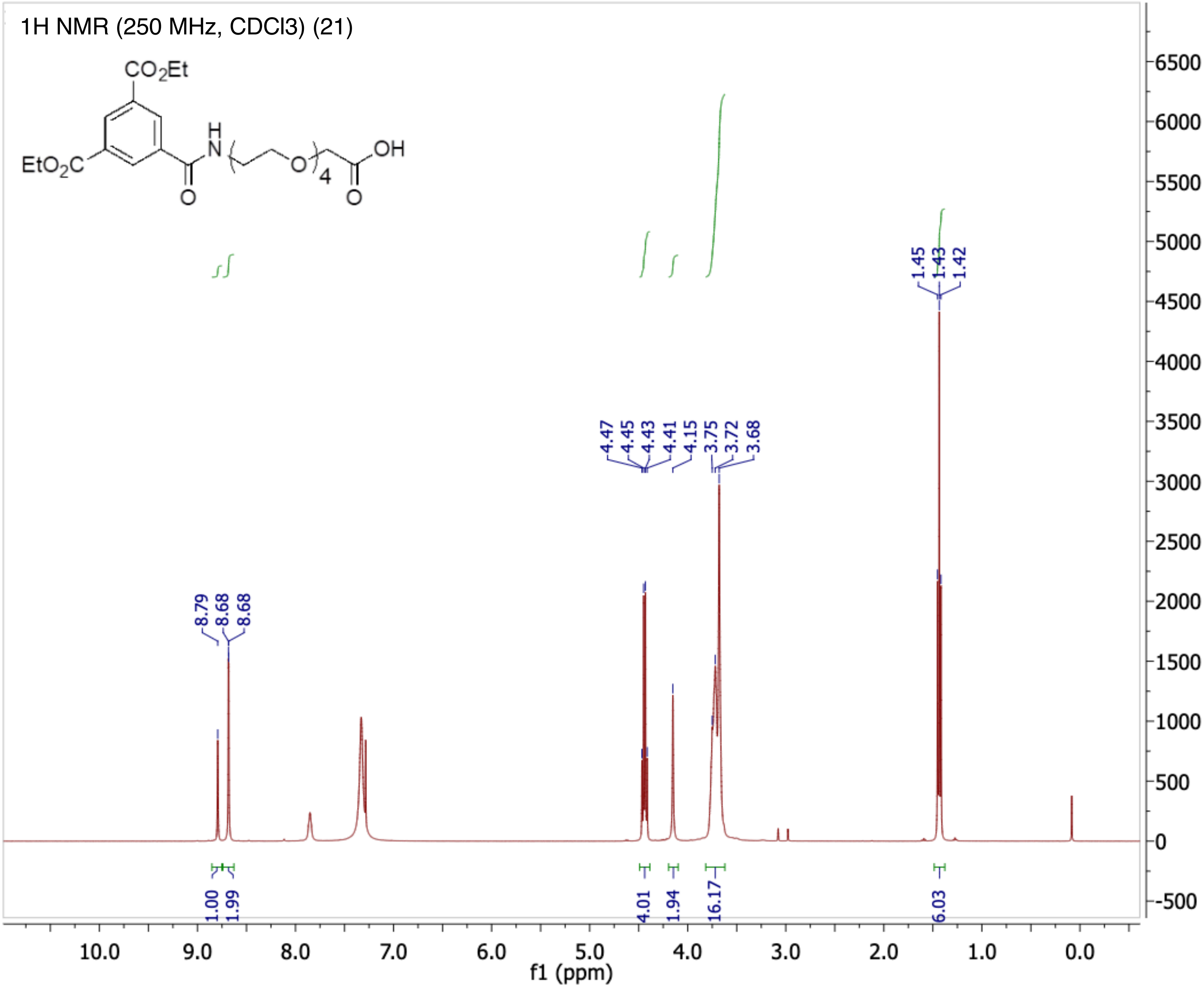
1H NMR (250 MHz, CDCl3) (21)
CO2Et
EtO2C
H
N
O
4
OH
O
O
8.79
8.68
8.68
4.47
4.45
4.43
4.41
4.15
3.75
3.72
3.68
1.45
1.43
1.42
1.00
1.99
4.01
1.94
16.17
6.03
f1 (ppm)

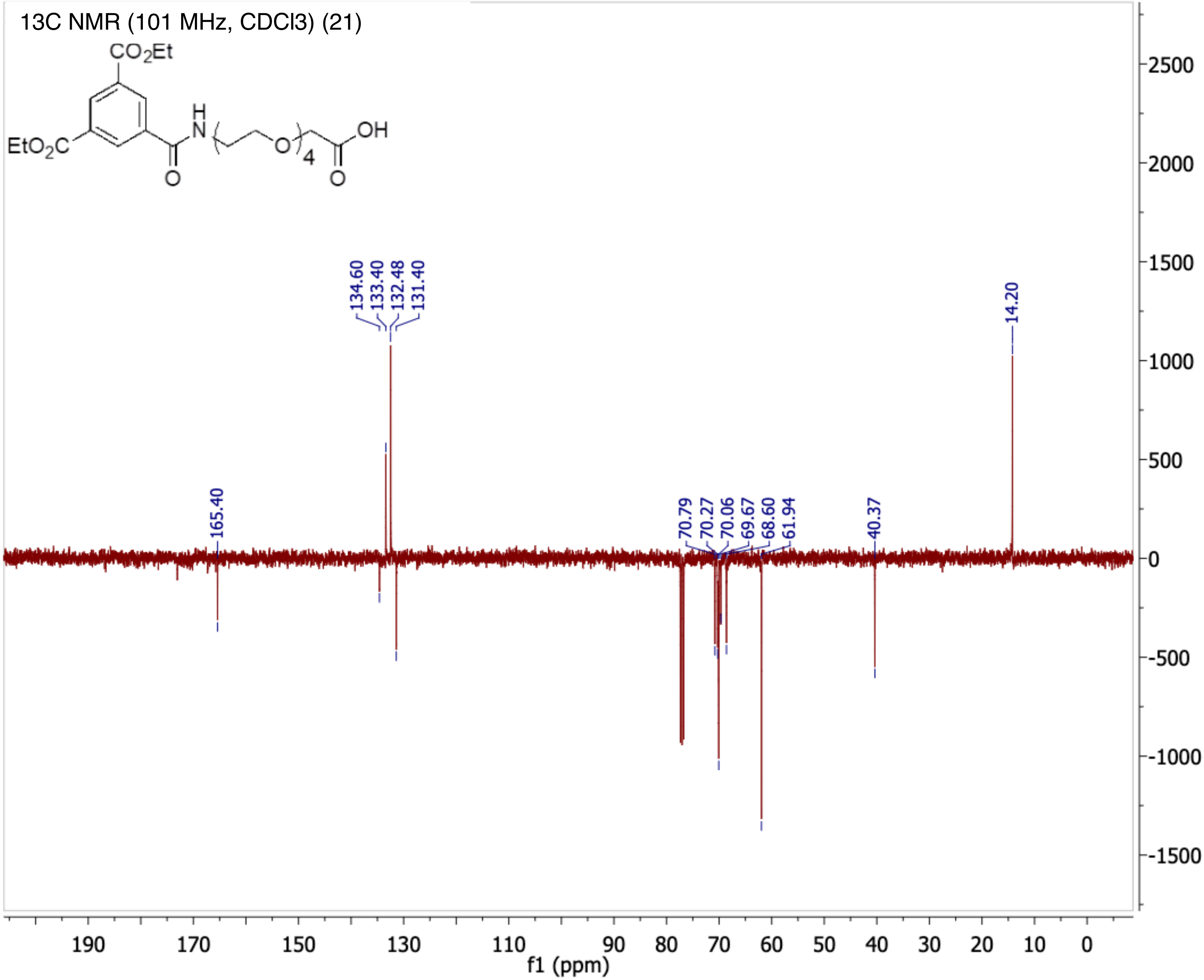
13C NMR (101 MHz, CDCl3) (21)
CO2Et
EtO2C
H
N
O
4
OH
O
O
134.60
133.40
132.48
131.40
14.20
165.40
70.79
70.27
70.06
69.67
68.60
61.94
40.37
2500
2000
1500
1000
500
0
-500
-1000
-1500
190
170
150
130
110
90
80
70
60
50
40
30
20
10
0
f1 (ppm)

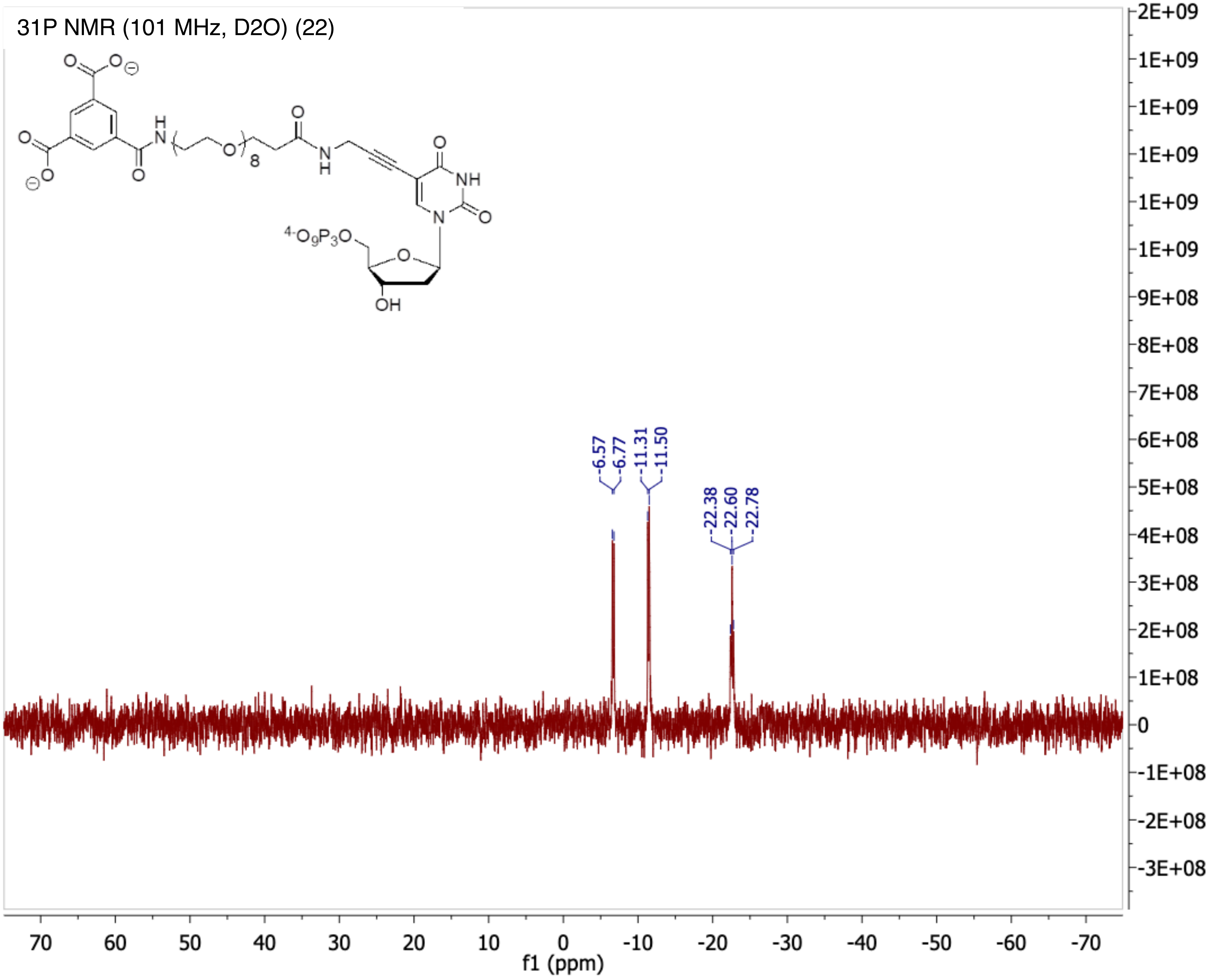

31P NMR (101 MHz, D2O) (22)
NH
N
OH
-6.57
-6.77
-11.31
-11.50
-22.38
-22.60
-22.78
f1 (ppm)

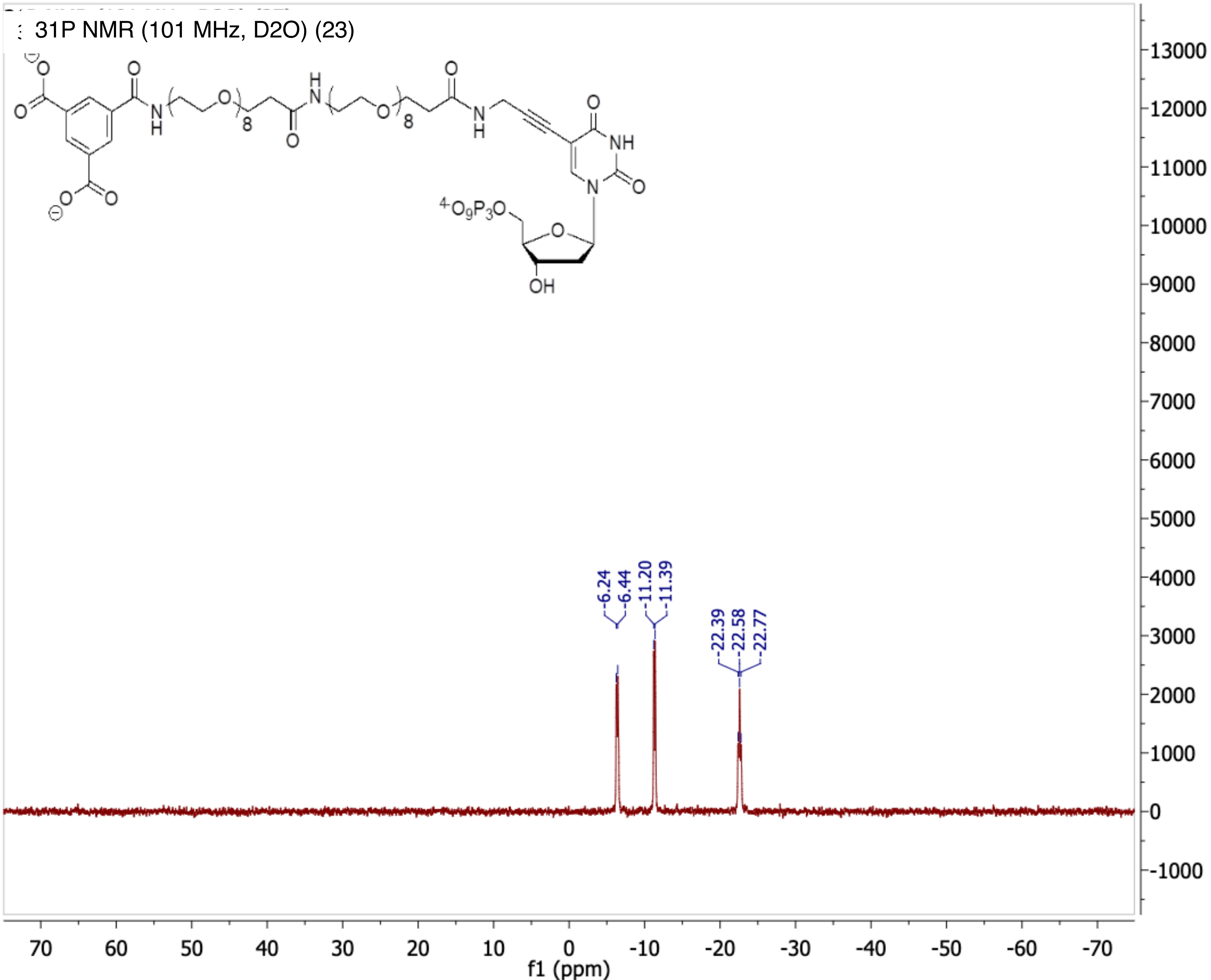
31P NMR (101 MHz, D2O) (23)
-6.24
-6.44
-11.20
-11.39
-22.39
-22.58
-22.77
f1 (ppm)

31P NMR (101 MHz, D2O) (24)

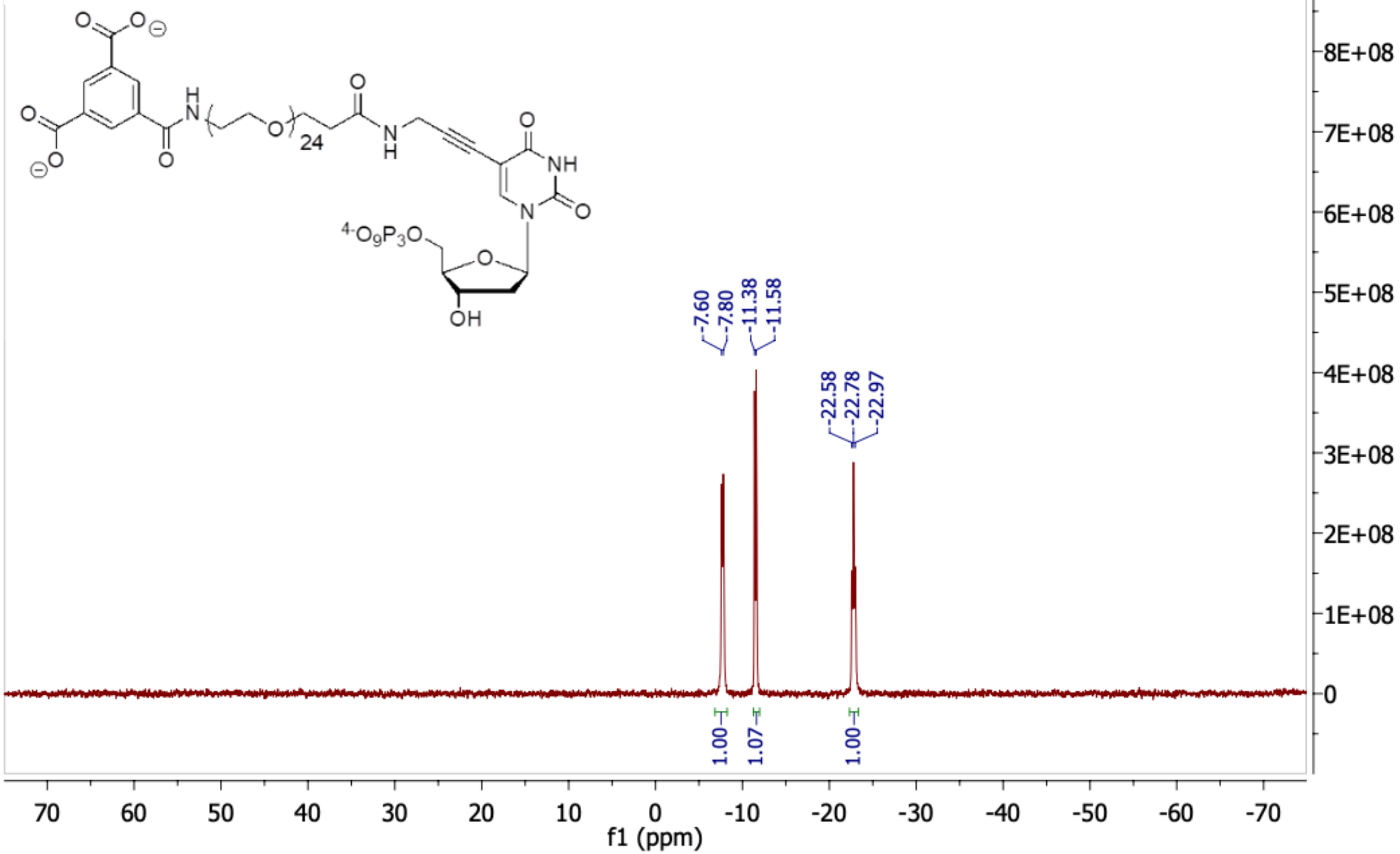

31P NMR (101 MHz, D2O) (25)

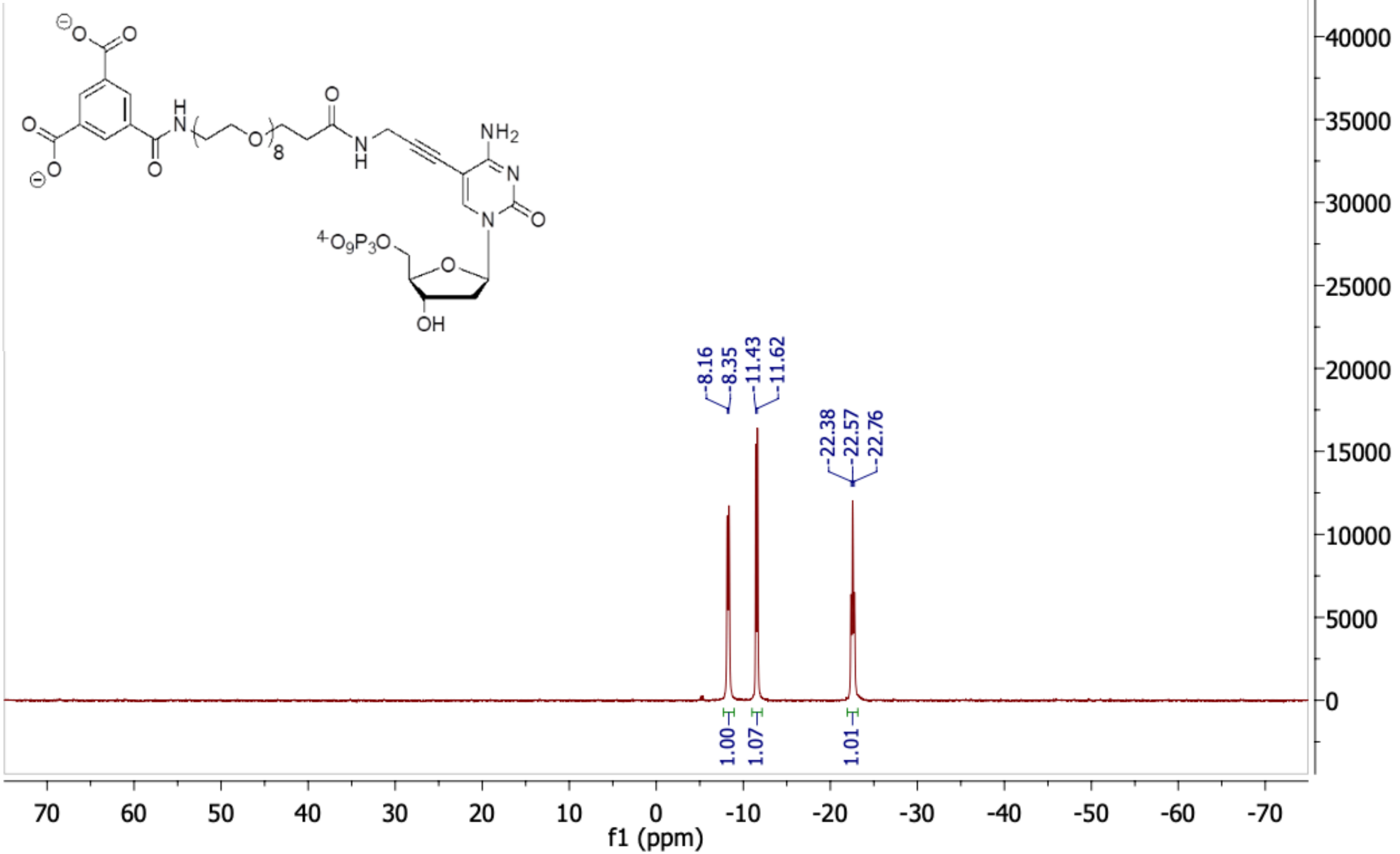

31P NMR (101 MHz, D2O) (26)

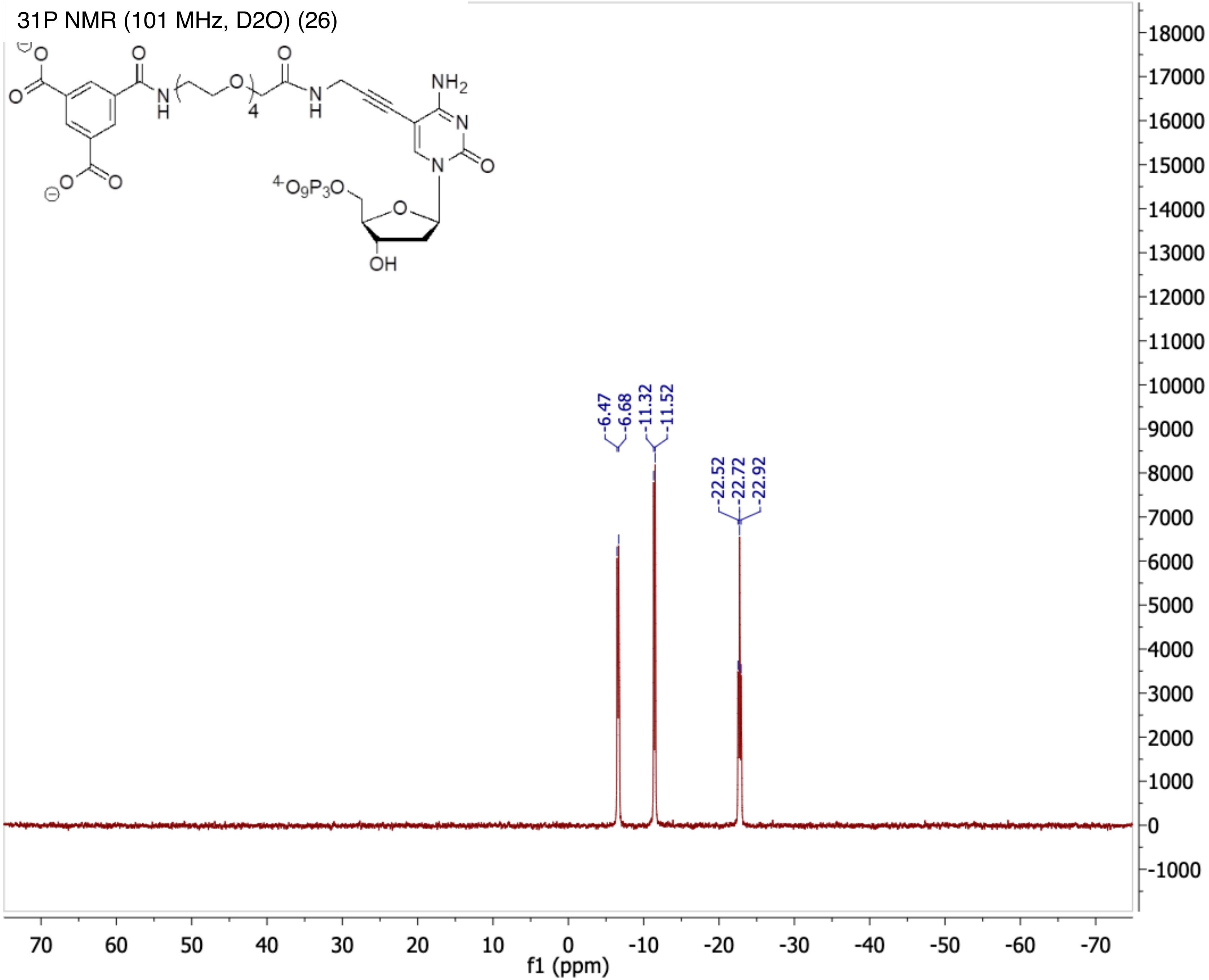

31P NMR (101 MHz, D2O) (27)

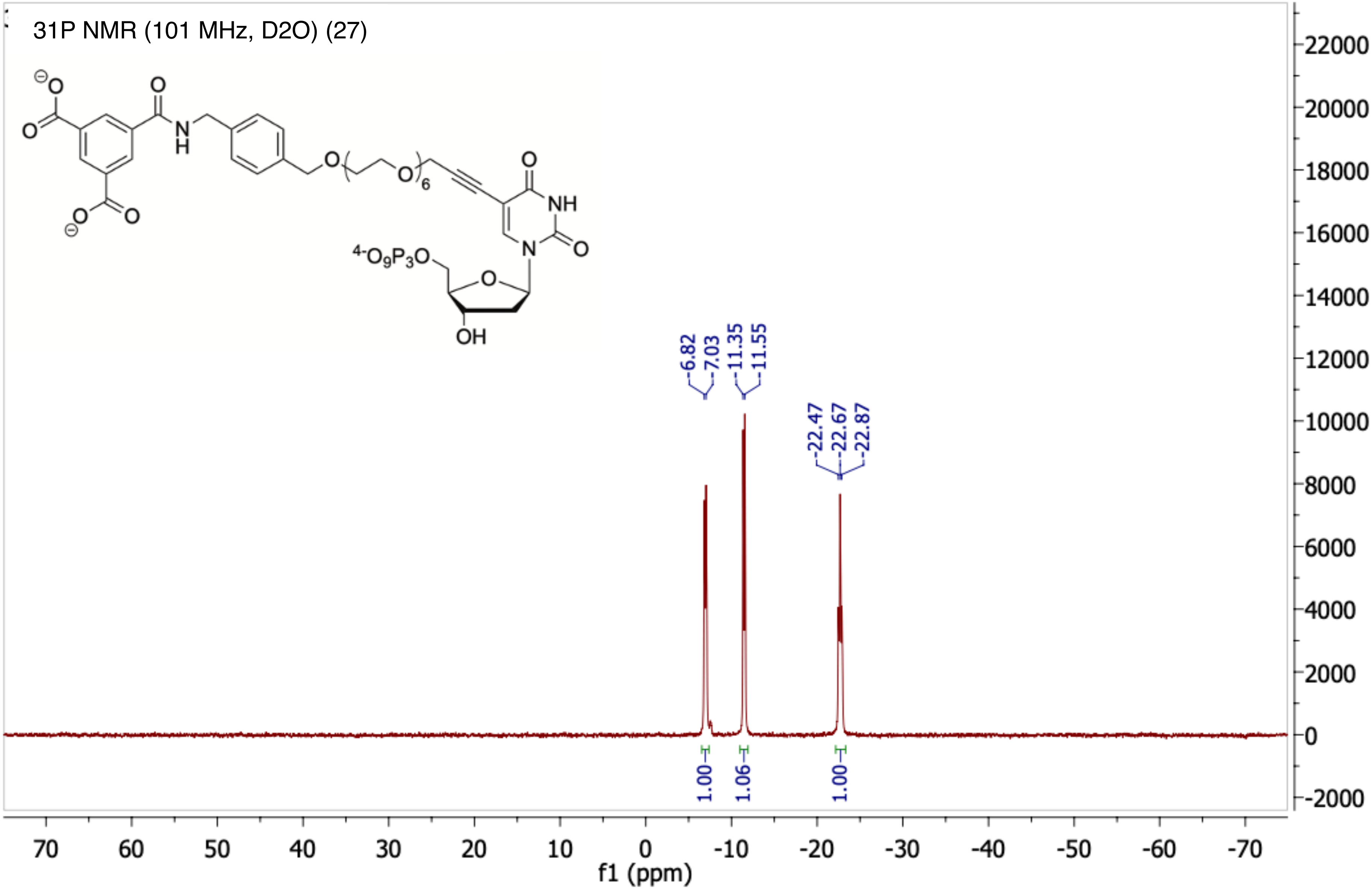

1H NMR (250 MHz, CDCl3) (28)

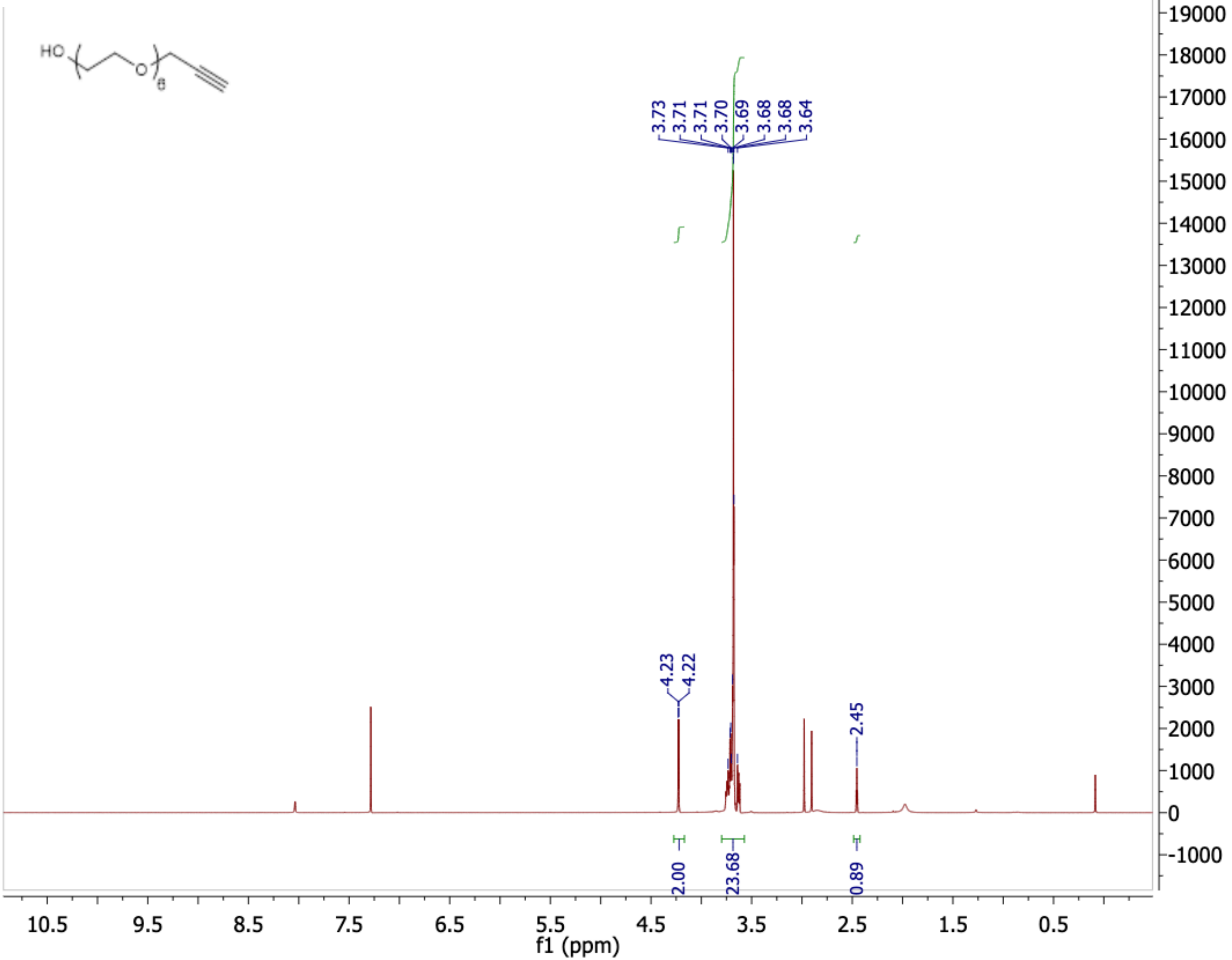

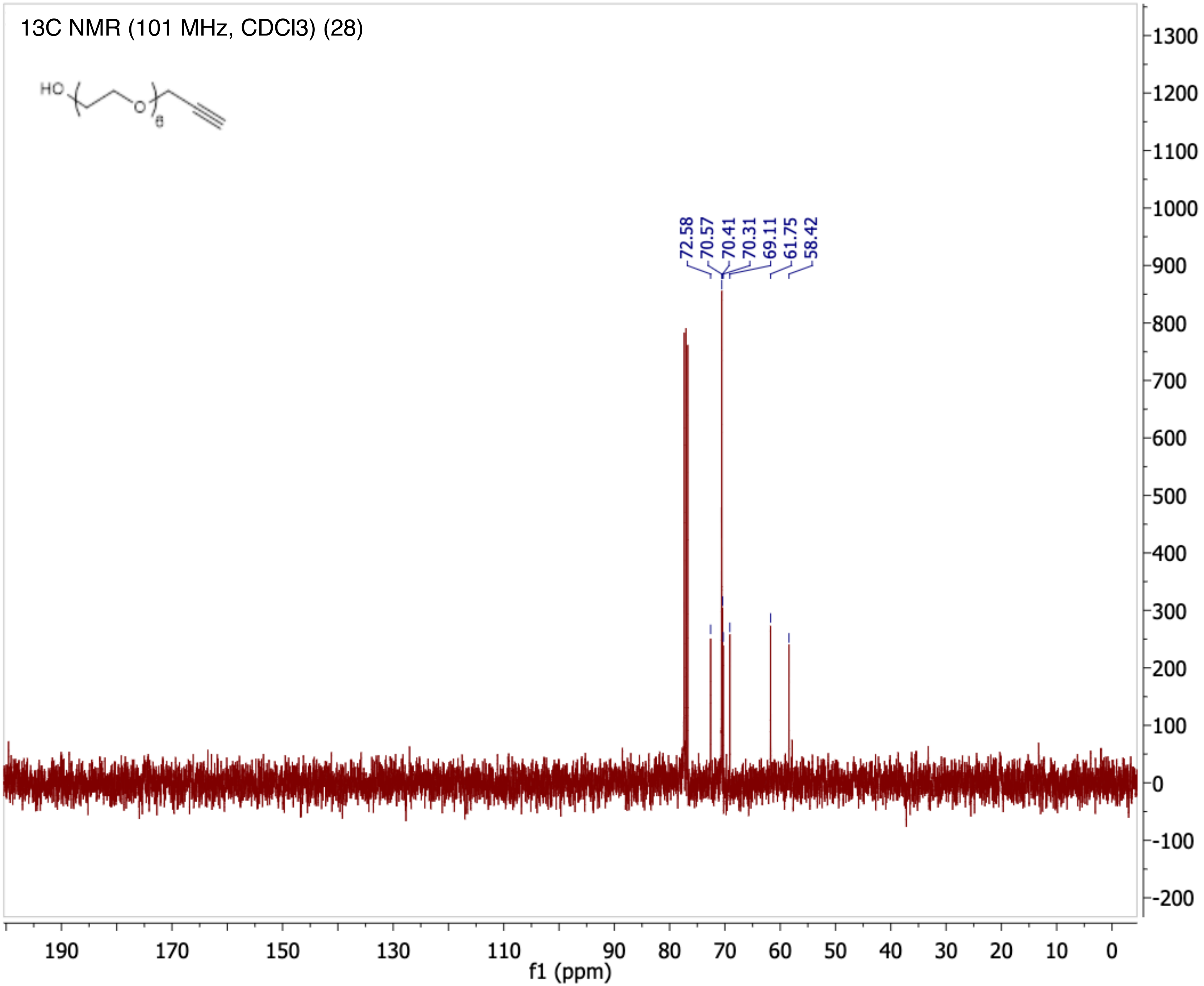
13C NMR (101 MHz, CDCl3) (28)
HO
O
8
72.58
70.57
70.41
70.31
69.11
61.75
58.42
f1 (ppm)

1H NMR (400 MHz, CDCl3) (29)

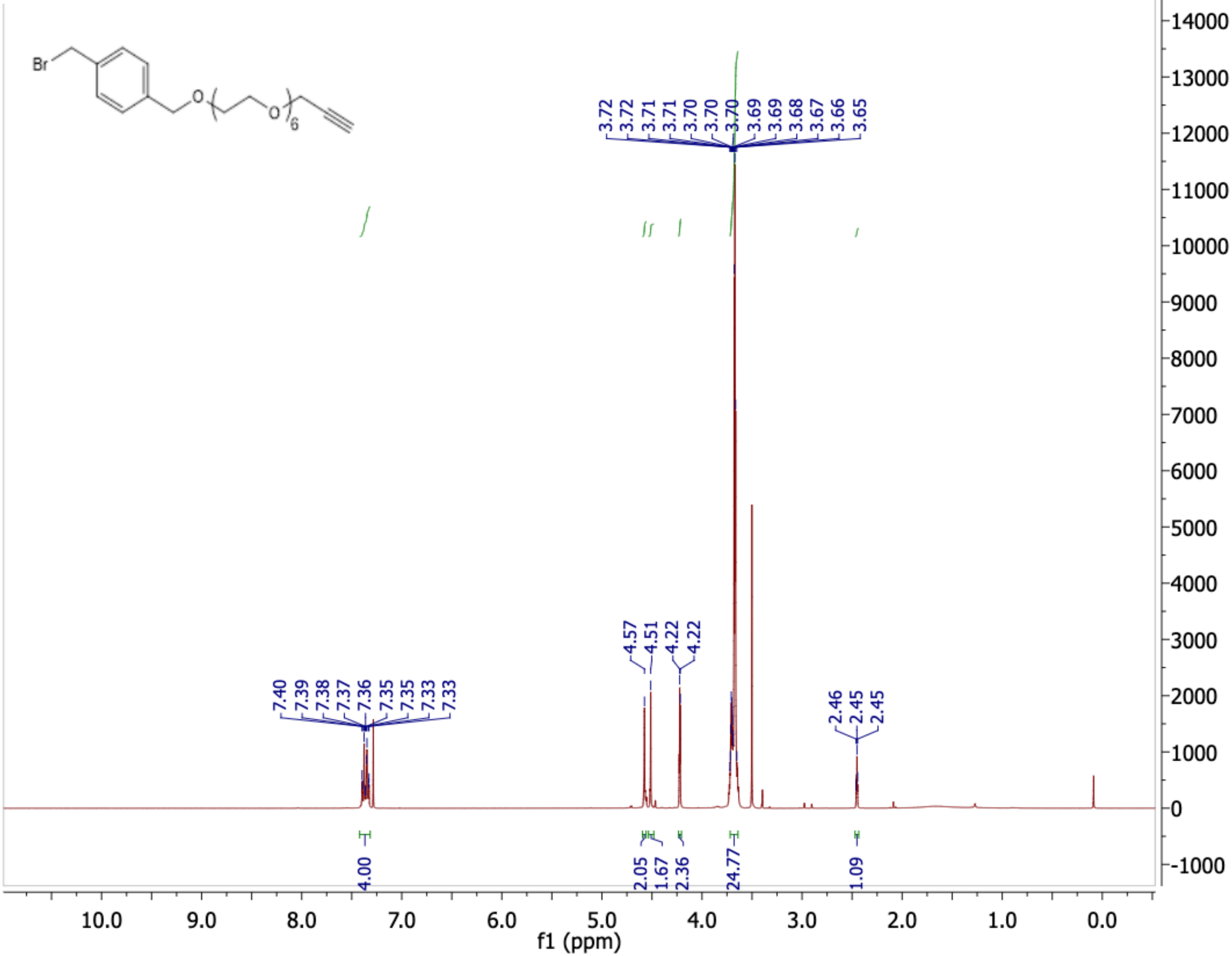

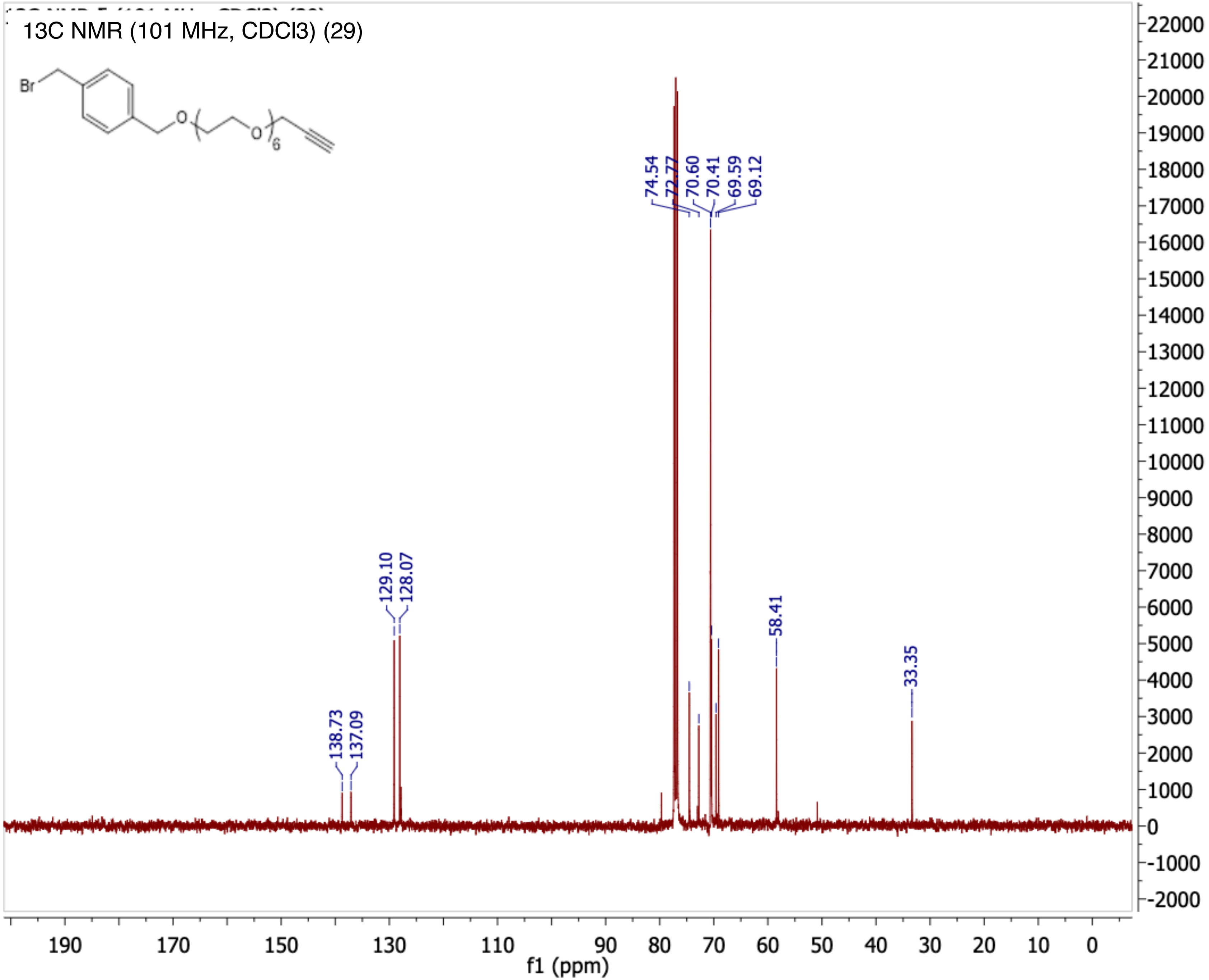

13C NMR (101 MHz, CDCl3) (29)
Br
O
O
6
74.54
72.77
70.60
70.41
69.59
69.12
129.10
128.07
138.73
137.09
58.41
33.35
f1 (ppm)

1H NMR (400 MHz, CDCl3) (30)

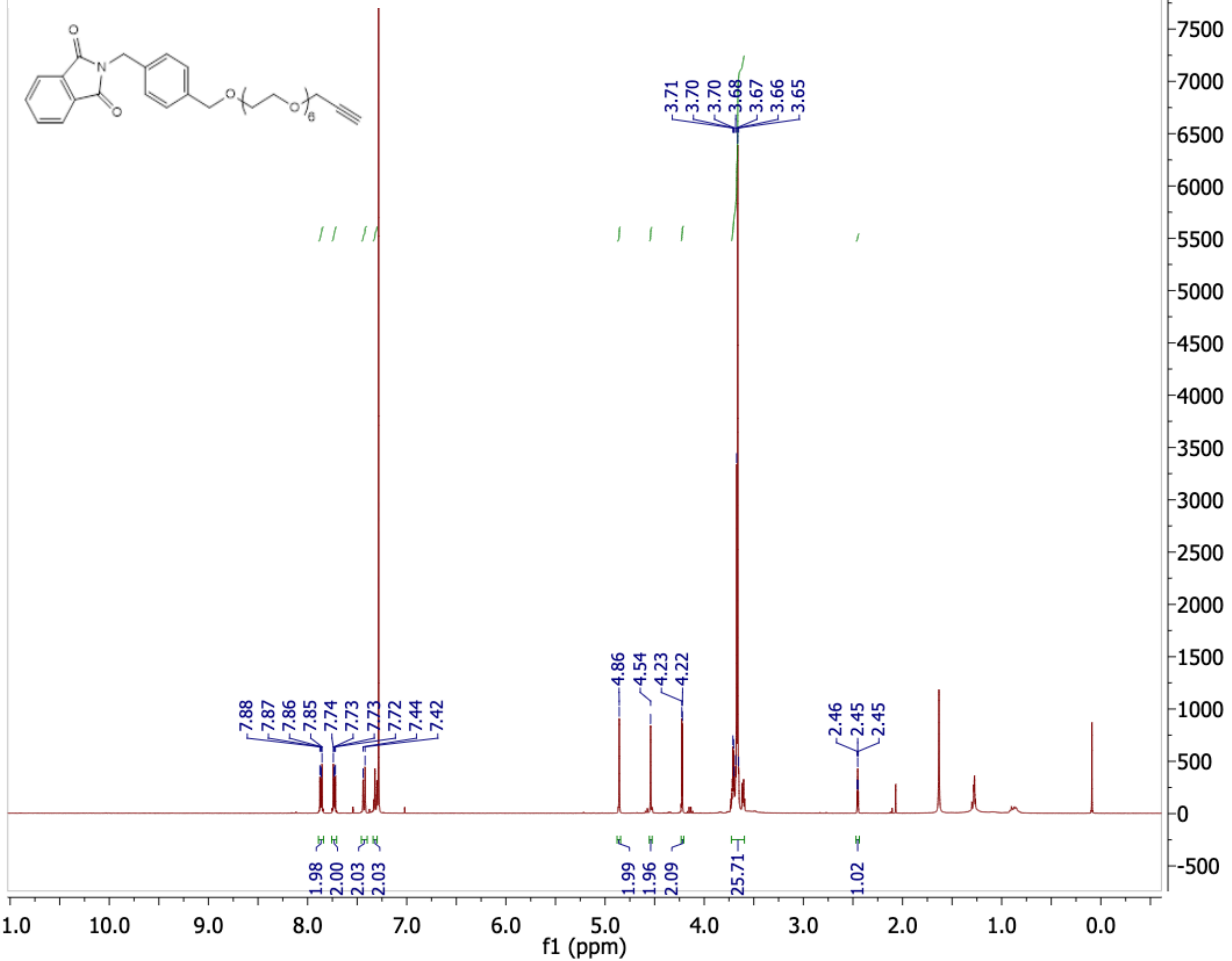

13C NMR (101 MHz, CDCl3) (30)

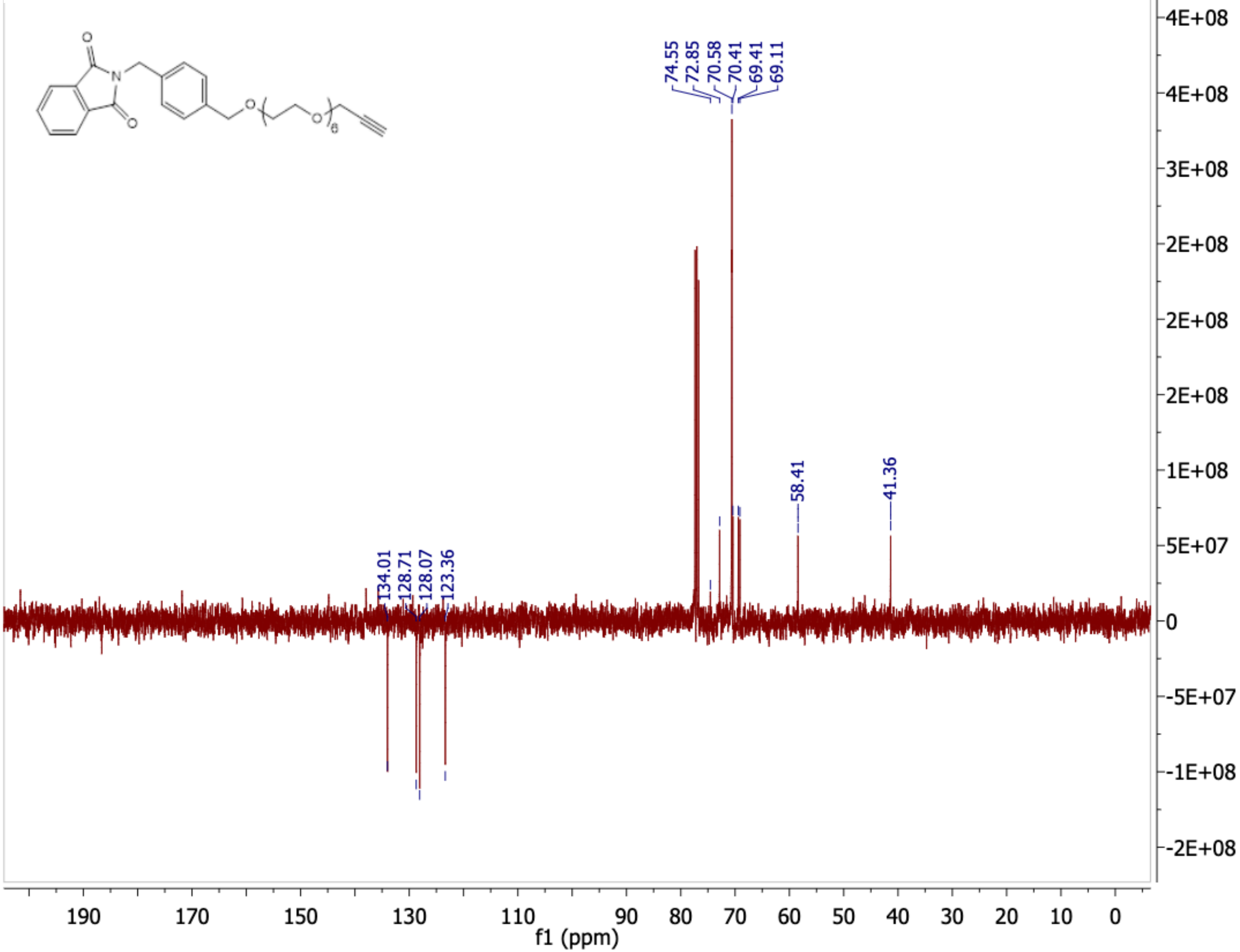

1H NMR (400 MHz, CDCl3) (32)

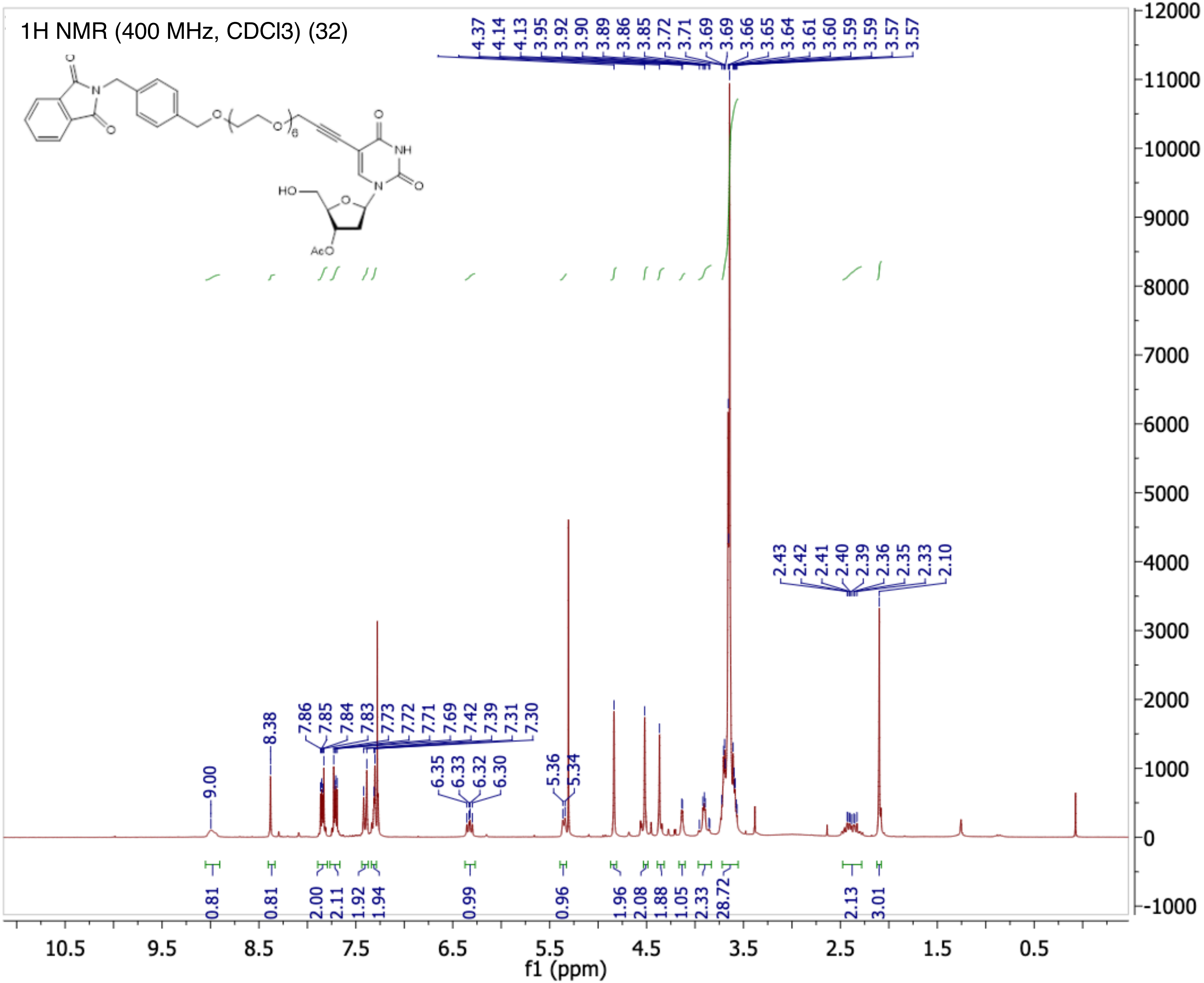

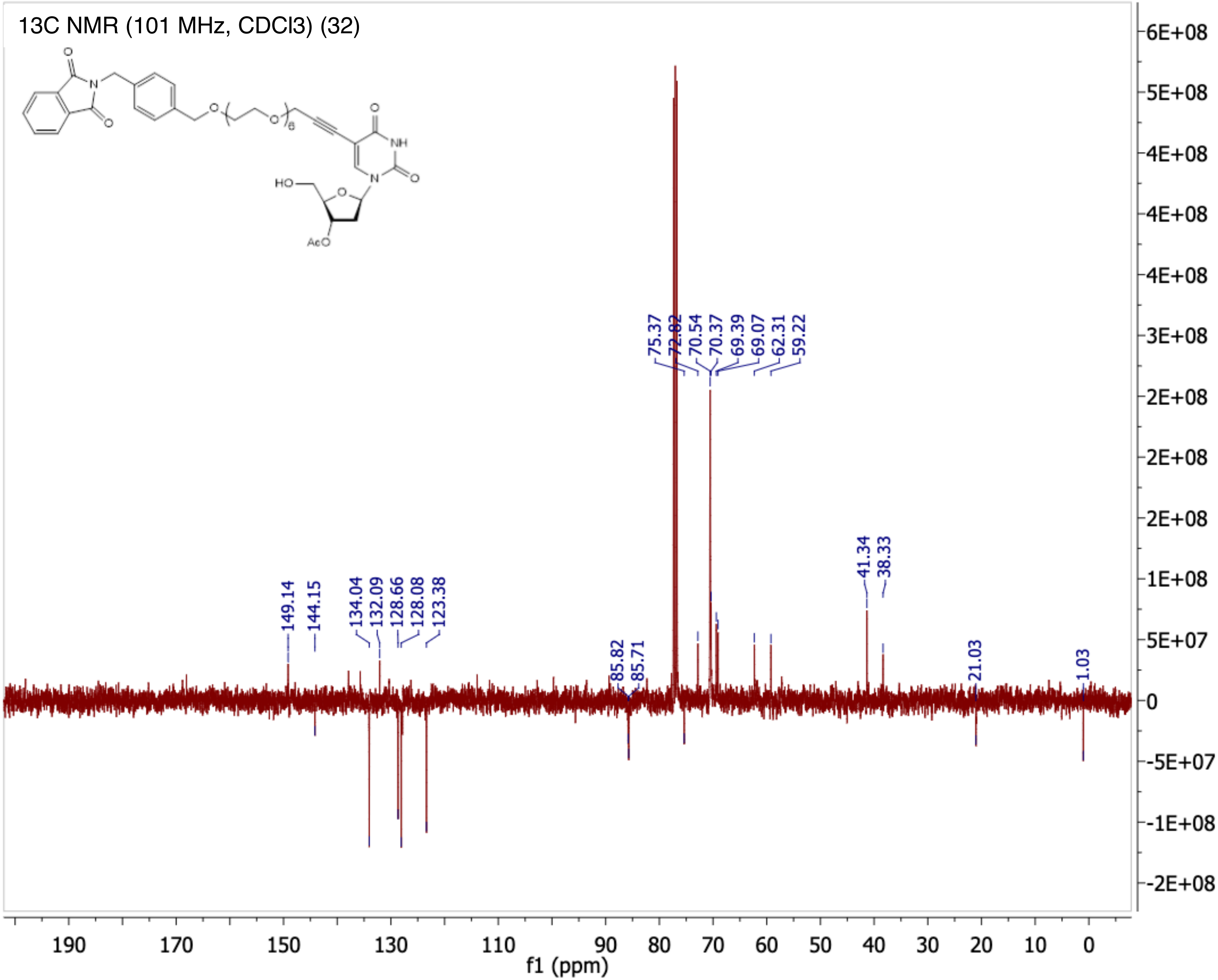
13C NMR (101 MHz, CDCl3) (32)
O
N
O
O
O
8
O
NH
N
O
HO
O
AcO
149.14
144.15
134.04
132.09
128.66
128.08
123.38
85.82
85.71
75.37
72.82
70.54
70.37
69.39
69.07
62.31
59.22
41.34
38.33
21.03
1.03
6E+08
5E+08
4E+08
4E+08
3E+08
2E+08
2E+08
2E+08
1E+08
5E+07
0
-5E+07
-1E+08
-2E+08
190
170
150
130
110
90
80
70
60
50
40
30
20
10
0
f1 (ppm)

31P NMR (101 MHz, D2O) (33)

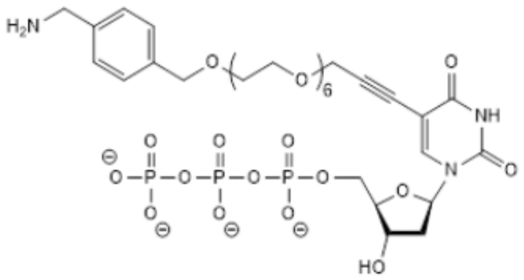

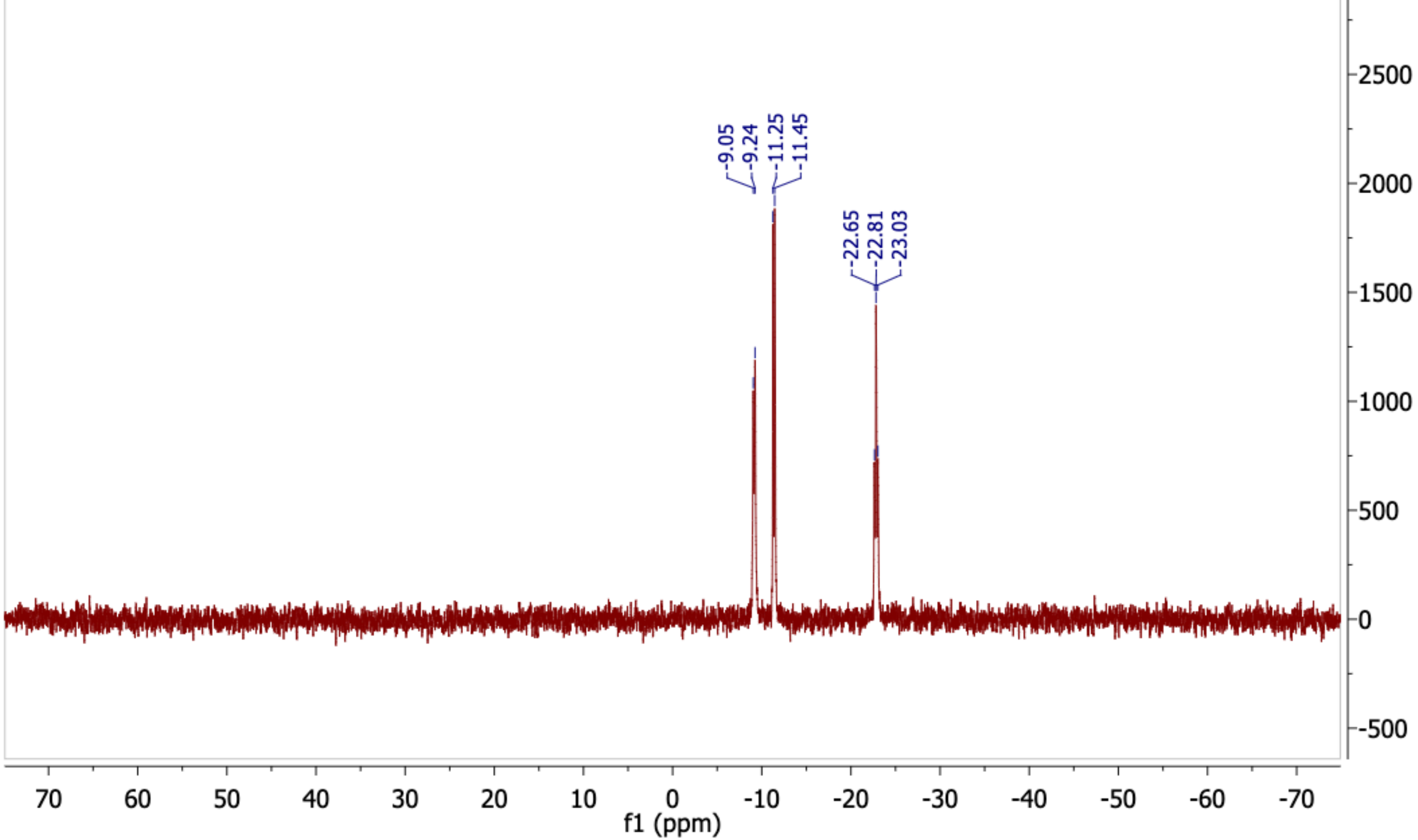

Supplement: Supplementary file 1 [file molecules-26-02250-s001.pdf]
